# Virus-Induced Changes of the Respiratory Tract Environment Promote Secondary Infections With *Streptococcus pneumoniae*

**DOI:** 10.3389/fcimb.2021.643326

**Published:** 2021-03-22

**Authors:** Vicky Sender, Karina Hentrich, Birgitta Henriques-Normark

**Affiliations:** ^1^ Department of Microbiology, Tumor and Cell Biology, Karolinska Institutet, Stockholm, Sweden; ^2^ Clinical Microbiology, Karolinska University Hospital, Solna, Sweden

**Keywords:** *Streptococcus pneumoniae*, pneumococci, influenza virus, COVID-19, respiratory tract infections, coinfection, influenza-pneumococcal coinfection

## Abstract

Secondary bacterial infections enhance the disease burden of influenza infections substantially. *Streptococcus pneumoniae* (the pneumococcus) plays a major role in the synergism between bacterial and viral pathogens, which is based on complex interactions between the pathogen and the host immune response. Here, we discuss mechanisms that drive the pathogenesis of a secondary pneumococcal infection after an influenza infection with a focus on how pneumococci senses and adapts to the influenza-modified environment. We briefly summarize what is known regarding secondary bacterial infection in relation to COVID-19 and highlight the need to improve our current strategies to prevent and treat viral bacterial coinfections.

## Introduction

The primary function of the respiratory system is to exchange oxygen and carbon dioxide by inhaling air. The average person inhales about 10,000 liters of air per day, which is laden with pollutants, allergens, and pathogens. The intake of contaminated air inevitably allows inhaled microorganisms to colonize the respiratory tract. One of the most commonly found bacterial pathogens in the respiratory tract is the gram-positive bacterium *Streptococcus pneumoniae* (the pneumococcus). The pneumococcus dynamically colonizes up to 30-75% of healthy children, especially those attending day care centers, as well as up to 20-30% of healthy adults (Ghaffar et al., 1999; Bogaert et al., 2004; Hjalmarsdottir et al., 2016; Lindstrand et al., 2016). Colonization is usually asymptomatic, but pneumococci can also spread into the lower respiratory tract to cause pneumonia, and to other sites of the body where it causes invasive diseases such as bacteremia and meningitis. Risk groups for developing a severe pneumococcal disease include young children and the elderly (<2 yrs and >65 yrs), immunodeficiencies, and comorbidities like diabetes. Also preceding virus infections constitute a major risk for developing severe pneumococcal diseases (Madhi et al., 2004). Pneumococci are most successful in causing disease, especially when risk factors are present, making them one of the leading causes of lower respiratory tract infections (LRTI) worldwide (GBD 2016 Lower Respiratory Infections Collaborators, 2018). Increased morbidity and mortality due to pneumococcal infections is closely linked to underlying virus infections, mainly caused by influenza virus (Morris et al., 2017).

The influenza virus causes a highly contagious respiratory illness, also known as the flu that is responsible for significant morbidity and mortality. Influenza-induced epidemics result in 3 - 5 million cases of severe illness, and up to 650 thousand deaths worldwide each year (World Health Organization, 2018). Besides the seasonal epidemics we witness every year, four influenza pandemics have occurred, since the beginning of the 20^th^ century: the Spanish influenza (H1N1) in 1918/1919, Asian influenza (H2N2) in 1957, Hong Kong influenza (H3N2) in 1968, and H1N1 swine influenza in 2009. Of these pandemic viruses, the 1918 virus was the most devastating resulting in 50 - 100 million deaths worldwide (Taubenberger and Morens, 2020). Many of the victims were rather young and secondary bacterial pneumonia, mainly caused by pneumococci, was a major cause of death among those infected with the virus (Morens et al., 2008). Also during the global outbreak of the H1N1 swine influenza in 2009, up to 34% of the fatal cases were associated with secondary bacterial infection, predominantly caused by *S. pneumoniae* (Centers for Disease Control and Prevention, 2009). Secondary pneumococcal infections occurring during or after a viral infection are often associated with negative outcomes. A combined infection of influenza and pneumococci can be either a coinfection or a secondary bacterial infection following influenza. Clinically, it is difficult to distinguish between a coinfection and a secondary pneumococcal infection and the term superinfection is commonly used for the incidence of a second infection superimposed on an earlier infection, often caused by a pathogen of different origin. In this review we mainly focus on secondary pneumococcal infection which is clinically more important and the unidirectional effects that the influenza virus has on pneumococcal disease are well-studied. However, research shows that the interaction is bidirectional and bacterial infection also affects the virus, which has been reviewed previously (Short et al., 2012).

In this review we summarize how a preceding influenza infection predisposes the host to secondary bacterial infection with pneumococci. We outline how virus-induced alterations of the pulmonary immune system promote a secondary bacterial infection with a focus on the two most common pathogens, influenza virus and pneumococci. We provide an overview of the recently emerging role of specific pneumococcal factors favoring secondary bacterial infection, and explain how bacterial sensing and adaptation in the virally modified environment contributes to disease severity. Finally, we summarize what is known about secondary bacterial infection in the current coronavirus disease 2019 (COVID-19) pandemic and highlight the need for development of new alternative therapies to prevent and treat viral-bacterial coinfections. The available data support the theory that influenza-induced modulation of host immune responses and the ability of pneumococci to sense and adapt to virus-modified environments drive the overwhelming severe lung infection.

## The Clinical Situation—Coinfections in CAP and HAP

Worldwide, lower respiratory tract infections are major causes of morbidity and mortality and are frequently caused by coinfecting pathogens. Coinfections are increasingly recognized as an underlying etiology to community-acquired pneumonia (CAP) and hospital-acquired pneumonia (HAP). Both the influenza virus and *Streptococcus pneumoniae* are among the most common causative agents of lower respiratory tract infections. The Global Burden of Disease study (GBD) 2017, estimated that lower respiratory tract infection caused by influenza accounted for 9,459 000 hospitalization and 145,000 deaths among all age groups with the highest mortality rate among adults older than 70 years (GBD 2017 Influenza Collaborators, 2019). In 2016, *Streptococcus pneumoniae* was identified as the leading cause of morbidity and mortality from lower respiratory infections globally, contributing to 1,189 937 deaths (GBD 2016 Lower Respiratory Infections Collaborators, 2018). The main age groups at risk are children younger than 5 years and adults older than 65 years. Improved molecular testing allows increased detection and thereby extends our epidemiologic understanding of coinfections. Treatment, however, is often limited or done as prevention without specific etiology. Identification of the etiologic agent promotes implications for infection prevention and control, and has important impacts for public health initiatives, such as encouragement for vaccination (Cawcutt and Kalil, 2017). Treatment of bacterial pneumonia relies on antibiotics and treatment of influenza infection on antivirals, and supportive care is often needed for hospitalized patients. Patients with CAP, showing symptoms of flu or are diagnosed with flu in the days or weeks before the onset of CAP, are often empirically treated with antibiotics and possibly antivirals. Such antibiotics target the most common pathogens causing the most severe secondary infections, like *S. pneumoniae* and *Staphylococcus aureus*, often as broad-spectrum antibiotics (Leekha et al., 2011). Antibiotic coverage for methicillin resistant *Staphylococcus aureus* can be initiated when patients have signs of necrotizing pneumonia, including rapid onset of acute respiratory distress or hemoptysis. However, the desired treatment needs to be tailored antibiotic treatment for specific bacterial pathogens isolated from blood or a high-quality sputum specimen (Chertow and Memoli, 2013). In 2019, WHO classified antibiotic resistance as one of the top ten threats to global health (World Health Organization, 2019). A recent study investigating the use of antibiotics in 76 countries over 15 years revealed that antimicrobial resistance is increasing worldwide (Klein et al., 2021), and a major driver of antibiotic resistance is overuse and misuse of antibiotics. The currently ongoing COVID-19 pandemic is linked to higher use of antibiotics which may lead to an increase of antibiotic resistance (Bengoechea and Bamford, 2020; Canton et al., 2020; Dona et al., 2020; Getahun et al., 2020; Murray, 2020). There is currently only few treatment options available for patients with viral infections who also get infected with multidrug resistant bacteria. This indicates the urgent need for developing new antimicrobial therapies to treat coinfections.

### Influenza-Induced Alterations of the Pulmonary Host Response

**Figure 1:**
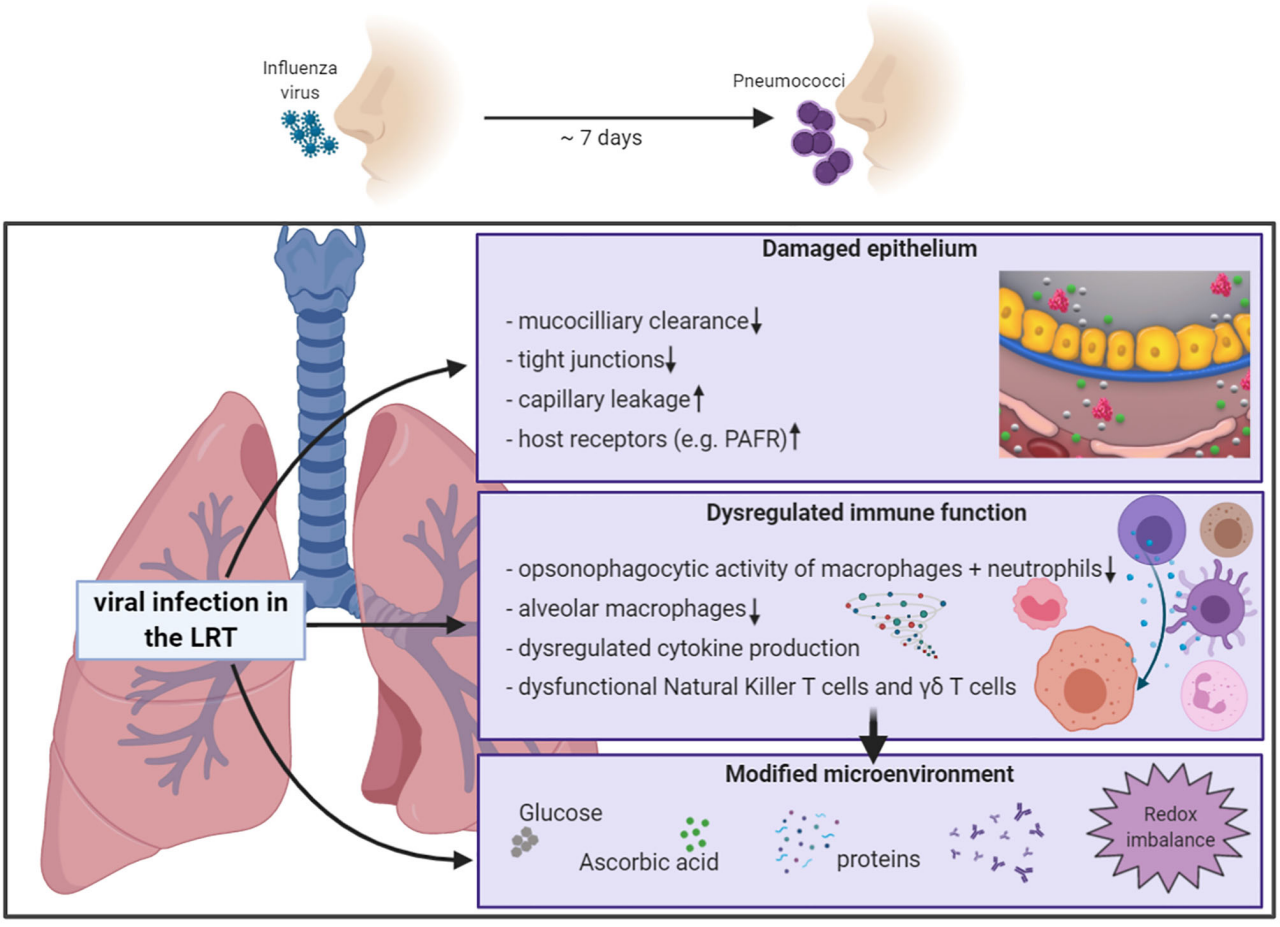
Schematic overview of influenza-induced alterations of the pulmonary host response. Increased sensitivity to secondary bacterial infection is partly mediated by influenza-induced effects on the pulmonary host response, including compromised epithelial barrier functions, innate and adaptive immune responses, and changes of the microenvironment in the respiratory tract. Partly adopted from Sender et al., 2020. *Created with BioRender.com*.

Increased morbidity and mortality from infections with influenza virus are often linked to bacterial superinfection. The complications associated with viral-bacterial coinfections are a result of altered host responses due to the virus infection (**Figure 1**). The innate immune response has a key role in protecting us against viral infections. Unfortunately, aspects of this immune reaction are also responsible for increased morbidity and mortality. We currently experience this from SARS-CoV-2 where interactions between the virus and immune cells lead to dysregulated immune responses, ultimately accelerating disease progression and severity, especially in older individuals (Guan et al., 2020; Huang et al., 2020; Qin et al., 2020; Zhang et al., 2020)

The lung epithelium covered with mucus provides the first line of defense against microbes entering the respiratory tract by brushing pathogens upwards through the mucociliary escalator. Once a virus successfully breaks the secretory mucus barrier, it invades epithelial cells to replicate. Influenza virus replication in the respiratory epithelium alters mucus production and reduces ciliary beating, which results in lower mucociliary clearance of pneumococci *in vivo*  (Pittet et al., 2010). In bronchial epithelial cells, the influenza virus reduces the secretion of Chitinase-3-like 1, a protein involved in anti-pneumococcal host response, and thereby promotes secondary pneumococcal infection (Dela Cruz et al., 2012; Karwelat et al., 2020). The influenza-induced epithelial damage exposes more attachment sites for bacteria, thus promoting invasion and severe disease (Plotkowski et al., 1986). Also, direct binding of influenza to pneumococci promotes adhesion to respiratory epithelial cells (Rowe et al., 2020). The influenza-induced tissue damage is greatest at around day 7 after infection which is the time where both humans and mice are most susceptible to secondary bacterial infection (Nugent and Pesanti, 1983). The 2009 H1N1 pandemic virus destroyed basal airway epithelial cells which affected lung repair mechanisms, thus explaining the high fatality rate of coinfections with this pandemic virus compared to the seasonal H1N1 virus (Kash et al., 2011). In addition to the direct effect on the airway epithelium, recruited inflammatory monocytes induce TRAIL-mediated lung damage, which facilitates pneumococcal invasion (Ellis et al., 2015). The activity of viral neuraminidases further promotes invasion by stripping sialic acids off the lung epithelium, which exposes adhesion receptors for pneumococci to bind (McCullers and Bartmess, 2003). Indeed, influenza A virus (IAV) infection increases the amount of the adhesion receptor platelet-activating factor (PAFR) (Van Der Sluijs et al., 2006b). However, neither mice deficient in PAFR nor PAFR antagonist treatment *in vivo* improved the outcome of secondary bacterial infections (McCullers and Rehg, 2002; Van der Sluijs et al., 2006b). Viral neuraminidase inhibitors only partially protect from bacterial complications following influenza virus infection (McCullers, 2004), and also, the use of neuraminidase treatment to inactive viruses does not affect the outcome of secondary bacterial infection in mice (Chockalingam et al., 2012). Thus, additional factors must play a role for the increased susceptibility to secondary pneumococcal infection, besides the influenza-mediated impact on the lung epithelium.

Phagocytic cells, including macrophages and neutrophils eliminate invading pathogens through opsonophagocytosis. We know that influenza virus infection suppresses the function of such phagocytic cells (Abramson et al., 1982; Debets-Ossenkopp et al., 1982; Astry and Jakab, 1984). Recent studies investigated how influenza virus infections affect the antibacterial activity of phagocytic cells in more detail. Sun & Metzger found that influenza-induced IFN-gamma impairs bacterial clearance by alveolar macrophages through downregulation of the class A scavenger receptor MARCO (Sun and Metzger, 2008). The scavenger receptor MARCO plays an important role in host defense against pneumococcal pneumonia (Arredouani et al., 2004). The antioxidant sulforaphane enhances MARCO expression and thereby improves pneumococcal clearance and host survival during secondary pneumococcal pneumonia (Wu et al., 2017). Similarly, IL-6 protects mice from secondary pneumococcal infection. Administration of recombinant IL-6 rescues macrophages from influenza-induced apoptosis and increases MARCO expression which promotes phagocytosis of bacteria (Gou et al., 2019). The functional impairment of alveolar macrophages allows noninvasive pneumococcal strains to cause deadly disease (Verma et al., 2020). In addition to functional departures, defects in antibacterial activity are also related to lower numbers of alveolar macrophages. The number of alveolar macrophages decreases to 85-90% compared with baseline levels, within 7 days after virus infection (Ghoneim et al., 2013; Smith et al., 2013). Also, dysfunctional neutrophils contribute to defects in antibacterial immunity during coinfection (Levine et al., 2001; McNamee and Harmsen, 2006). In coinfected lungs bacterial numbers remain high, despite the pro-inflammatory state with increased cytokines and more neutrophils (Levine et al., 2001). The reduced phagocytic activity of neutrophils is associated with higher expression of the inhibitory cytokine IL-10 (Van der Sluijs et al., 2004; Van der Sluijs et al., 2006a). Type I IFNs, which are essential for antiviral immunity during influenza infection (Muller et al., 1994), disrupt the migration of neutrophils, thus sensitizing the host for secondary bacterial infection (Shahangian et al., 2009; Nakamura et al., 2011). Considering the detrimental effects dysregulated cytokine production has on phagocytic cell function during influenza-pneumococcal coinfection, it is not surprising that also other key players of the cellular immune defense in the lungs are affected.

Dendritic cells bridge the innate to the adaptive immune response by producing cytokines and presenting antigens. Influenza-pneumococcal coinfection in dendritic cells synergistically upregulates pro-inflammatory cytokines, whereas anti-inflammatory cytokines, like IL-10, are downregulated by influenza, which might contribute to the immunopathology during coinfection (Wu et al., 2011). A study from our group showed that influenza-induced type I IFNs trigger the secretion of the pro-inflammatory cytokines IL-6 and IL-12 in dendritic cells (Kuri et al., 2013). However, co-infecting pathogens do not only affect the production and release of cytokine, but also modulate the expression and activation of pattern recognition receptors. W linked more IL-12p70 production during influenza infection to higher levels of Toll-like receptor (TLR) 3, which recognizes pneumococcal RNA, thus activating TRIF-dependent pro-inflammatory signaling in dendritic cells (Spelmink et al., 2016). Influenza and pneumococci also synergistically activate other Toll-like receptors (TLRs) and TLR-dependent signaling pathways, thus generating inflammation and promoting disease progression during coinfection (Karlstrom et al., 2011; Stegemann-Koniszewski et al., 2013; Rodriguez et al., 2019).

The preceding production of type I IFNs by the influenza virus also affects antibacterial T cell responses. An *in vivo* study in mice revealed that influenza-induced type I IFNs repress γδ T cell function and their production of IL-17 which is responsible for recruitment and activation of neutrophils. This is abrogated in mice lacking the IFN receptor and the adoptive transfer of γδ T cells from IFN receptor KO mice improves the pulmonary clearance of pneumococci in wild type mice (Li et al., 2012). This inhibitory effect of type I IFNs on IL-17 production by γδ T cells promotes secondary pneumococcal pneumonia by inhibiting neutrophil recruitment and thus bacterial clearance is mediated through type I IFN-dependent production of pulmonary IL-27 (Cao et al., 2014). T cell-derived IFN-gamma inhibits pneumococcal clearance by alveolar macrophages in influenza infected lungs (Sun and Metzger, 2008). Besides activation of antibacterial immunity, T cells also play a role in maintenance of tissue homeostasis and tissue repair and the tissue protective cytokine IL-22 limits secondary pneumococcal infection (Ivanov et al., 2013). CD8+ effector T cells produce the anti-inflammatory cytokine IL-10, thereby contributing to resolve lung inflammation during acute influenza infection (Sun et al., 2009). However, this regenerating response can lead to enhanced susceptibility to superinfecting bacterial pathogens. Indeed, the regeneration process creates a favorable environment for opportunistic pathogens like pneumococci, eventually resulting in pneumococcal superinfection.

The tight regulation of the pulmonary immune system that constantly balances pro- and anti-inflammatory signals to maintain immune homeostasis, can be disturbed by infections and especially polymicrobial infections. While synergistic immune activation by influenza and pneumococci generally leads to hyper-inflammation and tissue damage as described above, subsequent infection with these two pathogens can also result in desensitization. Alveolar macrophages isolated after a resolved influenza infection respond poorly to TLR stimuli, which prevents the initiation of antibacterial responses and allows outgrowth of bacteria such as pneumococci *in vivo* (Didierlaurent et al., 2008 *)*. The increased susceptibility to a pneumococcal infection after a primary influenza infection can last up to six weeks. Similarly, peripheral blood mononuclear cells isolated from influenza-infected patients show selective defects in the production of TNFα and IFNγ after stimulation with heat-killed pneumococci (Giamarellos-Bourboulis et al., 2009). Although only partly understood, TLR desensitization and inability to recruit effector cells might be caused by higher numbers of alternatively activated macrophages that support tissue repair and immune homeostasis, but that also suppress immune responses (Chen et al., 2012). The anti-inflammatory state during tissue repair and restoration of lung immune homeostasis involves multiple immune-suppressive mechanisms (Snelgrove et al., 2008; Hussell and Cavanagh, 2009). One example is the increased expression of the CD200 receptor for the negative regulatory ligand CD200 on myeloid cells during viral infections which raises the activation threshold for these cells to superinfecting bacteria, allowing pneumococcal outgrowth (Goulding et al., 2011). The imbalanced pulmonary immune homeostasis greatly contributes to the pathology during influenza-pneumococcal coinfection.

In view of the two major effects influenza infections have on the pulmonary immune response, hyper-inflammation and desensitization, it is important to keep a balance between immune and inflammatory mechanisms to minimize the damage of the lung tissue, while also ensuring adequate defense to infections by other pathogens. However, in addition to the effects on clearance and immune homeostasis, influenza infection also changes the environment in the lower respiratory tract (LRT). Pathogen adaptation to these changed conditions determines if the bacteria can survive, grow and successfully establish disease.

### Pneumococcal Growth and Adaptation in the Influenza-Infected Environment

**Figure 2:**
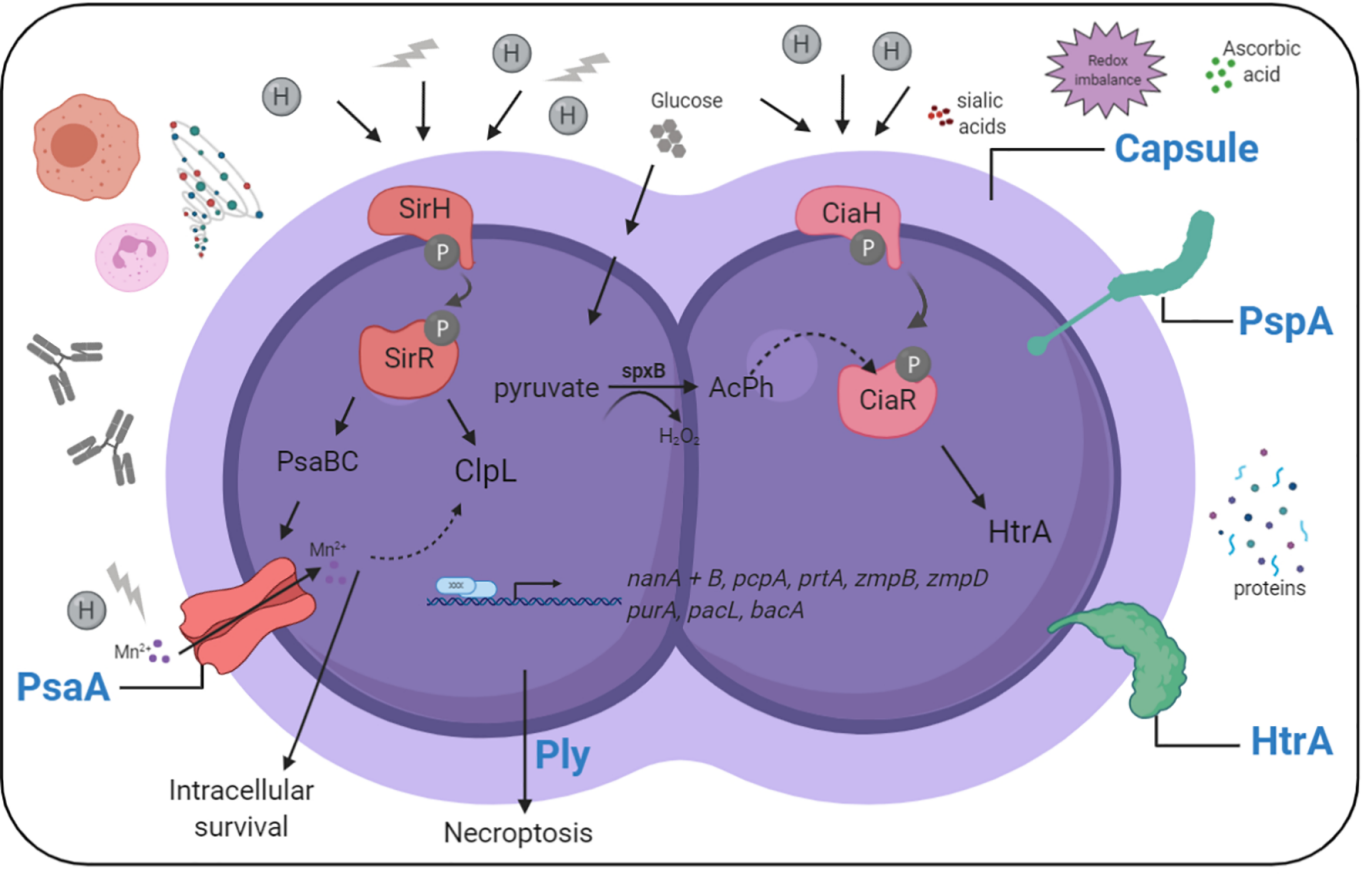
Simplified overview of pneumococcal sensing and adaptation in the influenza-infected respiratory tract. Pneumococci need to adapt to nutritional and environmental changes in the influenza-infected respiratory tract to cause disease. Sensing and adaptation of pneumococci in the influenza-infected respiratory tract includes activation of two component systems, and the expression of effector proteins helping the bacteria to grow and resist stress in this environment. *Created with BioRender.com*.

To survive during influenza-infected conditions in the respiratory tract, bacteria must adapt to the environment by increased expression and/or function of virulence determinants (**Figure 2** and **Table 1**). The role of bacterial factors and their implications in driving secondary bacterial pneumonia is only emerging recently. Some strains of pneumococci are more successful in causing disease after pre-infection with influenza than others. We know from *in vivo* studies in mice and ferrets, that the potential of pneumococci to cause severe disease during influenza coinfection, and to spread within a population, depends to a major part on the capsular serotype (McCullers et al., 2010). The pulmonary immune response during coinfections is pneumococcal strain-specific, where more virulent pneumococcal strains are associated with more severe secondary pneumonia (Sharma-Chawla et al., 2016). However, noninvasive strains can also become more invasive and cause lethal disease in influenza-infected mice (Verma et al., 2020). This suggests that additional, serotype-independent factors, contribute to the potential of the bacteria to cause disease.

**Table 1:** Pneumococcal virulence determinants and their effects on influenza-pneumococcal coinfection.

Pneumococcal virulence determinants	Effect on influenza-pneumococcal coinfection
Sequence type / serotype	Affects *in vivo* transmission in ferrets (Mccullers et al., 2010) Impacts pneumococcal virulence in mice (Mccullers et al., 2010; Sharma-Chawla et al., 2016)
-Carbohydrate transport and metabolism (Glucose, mannose, galactolitol) -Bacteriocins -Virulence factors (e.g. choline-binding protein A (PcpA), IgA proteases *zmpB* and *zmpD*)	Increased transcription in pneumococci from influenza-dispersed biofilms *in vitro* (Pettigrew et al., 2014)
Sialic acid metabolism and transport (Sialidase NanA, Sialic acid transporter SatABC)	Higher *NanA* transcription in bacteria from influenza-dispersed biofilms (Pettigrew et al., 2014), higher bacterial loads in i*n vivo* mouse models of colonization and otitis media, better adherence to epithelial cells *in vitro* (Wren et al., 2017 *)* No effect of *nanA* deletion in a mouse pneumonia model (King et al., 2009) Enhanced bacterial load in presence of the main sialic acid transporter SatABC (+/- sialidases NanA and NanB) in a mouse pneumonia model (Siegel et al., 2014)
Pneumococcal surface protein A PspA	Increased virulence in a mouse pneumonia model (King and Harmsen, 2009), higher transcription in pneumococci isolated from influenza-dispersed biofilms (Pettigrew et al., 2014)
High temperature requirement A HtrA	Increased bacterial load in a mouse pneumonia model (Sender et al., 2020)
Pneumolysin Ply	Contributes to necroptosis and virulence in epithelial cells *in vitro* and in a mouse pneumonia model (Gonzalez-Juarbe et al., 2020) No effect on virulence *in vitro* and *in vivo* using CRISPRi-Seq (Liu et al., 2020)
-Adenylsuccinate synthetase PurA capsular operon -Calcium-transporting ATPase pacL bacA	Increased pneumococcal virulence in a mouse pneumonia model (Liu et al., 2020)
Two-component system SirRH and ClpL and PsaB	Higher pneumococcal survival in influenza-infected epithelial cells *in vitro* (Reinoso-Vizcaino et al., 2020)
Two-component system CiaRH	Increased bacterial load in a mouse model (Sender et al., 2020)

We are only beginning to understand the role of specific pneumococcal factors in secondary bacterial infection. Transcriptional changes of pneumococci dispersed from influenza-induced biofilms suggest that many factors and adaptive changes help pneumococci to survive and thrive in influenza-infected conditions (Pettigrew et al., 2014). The upregulated genes indicate that bacteria adapt to changes of nutrients and stress. High presences of the Neuraminidase A (NanA), NanB and PTS transporters for rapid uptake of carbohydrates, such as mannose/fructose, glucose, and galactitol, demonstrate a clear link to carbohydrate metabolism. Virulence factors that are increased in pneumococci recovered from influenza-dispersed biofilms include *nanB*, *pcpA*, *pspA*, *prtA*, and the IgA proteases *zmpB* and *zmpD* (Pettigrew et al., 2014). Pneumococci depend on carbohydrates as a carbon source (Buckwalter and King, 2012). Sialic acids are one such carbon source utilized by pneumococci (Marion et al., 2011). Intrinsic neuraminidase activity releases sialic acids which promote pneumococcal growth (Burnaugh et al., 2008) and serve as a signal that increases virulence (Trappetti et al., 2009). During coinfections, pneumococci feed on influenza-provided sialic acids which promotes colonization and development of pneumonia through aspiration (Siegel et al., 2014). However, the activity of viral neuraminidases is insufficient to fully compensate for the absence of NanA in pneumococci, suggesting an important role for NanA properties other than its enzymatic activity in pneumococcal pathogenesis (Wren et al., 2017). Interestingly, another study observed that NanA was dispensable for pneumococcal outgrowth during coinfection (King et al., 2009), which can be explained by the niche-specific expression patterns of NanA, with more NanA in the nasopharynx than in the lungs (Lemessurier et al., 2006). The overall data indicate that the specific conditions in the local environment determine which genes/proteins are induced to achieve an advantage for pneumococci in just that specific conditions at that time.

The LRT provides, especially during influenza infection, additional nutrients such as glucose, which leaks into the lungs from the blood (Sender et al., 2020). Glucose is the preferred carbon source for pneumococci and glucose-mediated catabolite repression further explains why sialic acid-dependent growth is more important in the nasopharynx. This study also shows that not only glucose, but also antioxidants derived from influenza-induced inflammation and capillary leakage allow pneumococcal outgrowth in the LRT (Sender et al., 2020). We describe influenza-induced redox imbalances in the LRT to which pneumococci adapt by inducing the pneumococcal surface protease/chaperone high temperature requirement A (HtrA), that helps the bacteria to grow under oxidative stress condition *in vitro* and *in vivo*, and protects them from host-mediated opsonophagocytosis by maintaining capsular production (Sender et al., 2020). Hemoglobin, the iron-containing metalloprotein in erythrocytes also supports pneumococcal growth *in vitro* and enhances the ability of pneumococci to feed on host glycoproteins, providing an advantage during colonization and infection (Akhter et al., 2020), especially under influenza-infected conditions where host hemoproteins may be available in the lungs. These studies highlight the ability of pneumococci to adapt to nutritional changes and stressors during influenza infection and imply that complex bacterial adaptation to multiple site- and time-specific changes plays a key role for the development of severe pneumococcal infection.

In the LRT of influenza-infected mice, we found increased cytokines, more immune cells, more antimicrobial peptides, and high levels of plasma proteins (Sender et al., 2020), suggesting that pneumococcal clearance would be promoted in this location. However, the high numbers of proteins present in the lower airways in the highly oxidizing environment during influenza infection likely also induces membrane stress for pneumococci, which the protease HtrA can help to reduce by digesting denatured proteins (Cassone et al., 2012). Another study showed that membrane stress induced by the antimicrobial peptide LL-37 leads to cell surface accumulation of HtrA (Mucke et al., 2020). The major environmental changes pneumococci need to adapt to in influenza-infected conditions include nutritional changes and oxidative stress, which affect surface protein expression.

Influenza-induced oxidative stress also promotes necroptosis caused by the pneumococcal cytotoxin, pneumolysin (ply) (Gonzalez-Juarbe et al., 2020). Necroptosis is a form of regulated inflammatory cell death which can be induced by both influenza infection (Nogusa et al., 2016) and the pore-forming toxin, ply, of pneumococci (Gonzalez-Juarbe et al., 2015), resulting in release of molecules that enhance pro-inflammatory processes and viral clearance in the lungs, but can also disrupt immune homeostasis. The study of Gonzalez-Juarbe et al., 2020 investigated the role of necroptosis during influenza-pneumococcal coinfection and in a series of experiments they show that influenza-induced necroptosis can be inhibited by antioxidant treatment, resulting in reduced disease severity and less tissue damage during secondary pneumococcal infection (Gonzalez-Juarbe et al., 2020). A potential advantageous effect of antioxidant treatment for pneumococci themselves and pneumococcal growth, as observed in our study (Sender et al., 2020), was not investigated in this study. However, antioxidant treatment was performed at 12 and 24 hrs after bacterial infection, whereas our study focused on early bacterial growth between 4-6 hrs after pneumococcal infection. The question remains if antioxidant treatment during coinfection is beneficial or detrimental and it might depend on timing, delivery route and dose. The role of ply in coinfection is controversial. Whereas the previous study suggests that ply contributes to mortality during coinfection, another study did not find any difference in a coinfection of wt and ply-lacking pneumococci (Gonzalez-Juarbe et al., 2020; Liu et al., 2020). However, these discrepancies can be due to variations in the coinfection model (such as C57BL/6 vs BALB/c mice and day 5 vs day 7 after virus infection) and the pneumococcal strains used (TIGR4 vs D39). The latter study also identified *purA*, the capsule operon, pacL and *bacA*, as essential genes for pneumococcal growth during influenza infection when compared to *in vitro* growth in C+Y medium (Liu et al., 2020). The authors confirmed the importance of the capsule gene locus in an *in vivo* coinfection model. This supports the data from our study where pneumococci lacking HtrA were phagocytosed more due to lower capsule production, indicating that the capsule is indeed an important virulence factor during coinfection (Sender et al., 2020). This underlines the concept that rapid bacterial clearance is a major factor influencing the severity of coinfections, which can be disturbed by the influenza-mediated dysfunction of major phagocytic cells and by bacterial adaptation to inflammatory environments.

Another virulence factor that interferes with host-mediated bacterial killing and also contributes to bacterial outgrowth during secondary pneumococcal infection in mice, is the pneumococcal surface protein A (PspA). Immunization with PspA reduces the bacterial load in the lungs early during coinfection (King et al., 2009). This demonstrates that our constantly increasing knowledge regarding the role of specific pneumococcal proteins and a better understanding of how they contribute to severe secondary pneumonia, will help us to develop alternative treatment options.

### Pneumococcal Sensing in the Influenza-Infected Environment

It is evident that pneumococci need to adapt to nutritional and environmental changes in the influenza-infected respiratory tract to cause disease. Transcriptomic analyses, combined with *in vivo* experiments using pneumococci with specific gene deletion, convincingly demonstrate the importance of certain genes/proteins for pneumococci during coinfection (**Figure 2** and **Table 1**). However, how exactly influenza-modified environments enable different bacterial factors to promote disease is a recently emerging field.

Pneumococci sense and respond to environmental changes with the help of two component systems (TCS), which consist of a membrane-bound histidine kinase (HK) that is autophosphorylated when sensing a signal, and transfers phosphate to a cytoplasmatic response regulator (RR), then acting as a transcriptional regulator (Stock et al., 1989). Pneumococci possess 13 TCSs and a single RR of which several are associated with virulence regulation (Throup et al., 2000). TCS1, also known as SirRH, senses influenza-induced acidic and oxidative stress, and controls pneumococcal adaptation *via* induction of *clpL* and *psaB*, which are required for intracellular survival of pneumococci (Reinoso-Vizcaino et al., 2020). In our study, TCS05, also known as CiaRH, induces *htrA* under influenza-infected conditions which helps the bacteria to cope with oxidative stress on their cell surface and protects them from host-mediated killing (Sender et al., 2020). CiaR phosphorylation, and hence *htrA* induction, can also be accomplished by internal acetyl phosphate generated by SpxB oxidation of pyruvate (Pericone et al., 2003; Hentrich et al., 2016). Free sialic acids, which we found to be increased in influenza-infected conditions, are taken up by pneumococci and converted by the SpxB pyruvate oxidase to acetyl-phosphate and hydrogen peroxide, which allows transcriptional activation of the *htrA* promoter *via* phosphorylated CiaR (Hentrich et al., 2016).

In mice and most other mammals, the dominating sialic acid is N-glycolylneuraminic acid (Neu5Gc) whereas in humans, due to a mutation in the *CMAH* gene, N-acetylneuraminic acid (Neu5Ac) decorates the glycan chains (Chou et al., 1998; Muchmore et al., 1998). The pneumococcus has higher transcription of *htrA* and *nanA* and increased sialidase activity in response to human-like Neu5Ac as compared with Neu5Gc (Parker et al., 2012; Hentrich et al., 2016; McCombs and Kohler, 2016), suggesting a specific pneumococcal adaptation to the virally inflamed human LRT where the synergistic activity of viral and bacterial neuraminidases contributes to the pathology of viral-bacterial coinfection.

### Similarities and Differences Between Influenza A Virus (IAV) and Other Respiratory Viruses With a Focus on SARS-CoV-2

Neuraminidase (NA) and hemagglutinin (HA) are the two major glycoproteins present on the surface of IAV, and they interact with the host sialic acids to invade cells and replicate (Gottschalk, 1958). The different forms of these surface glycoproteins determine the influenza virus subtype. To date, 16 HA (H1-16) and 9 NA (N1-9) subtypes have been identified in birds (McAuley et al., 2019). The subtype H1N1 and H3N2 are endemic in humans, circulating constantly within the population and cause seasonal outbreaks. Subtypes H5N1, H7N9 and H9N2 occasionally occur *via* zoonotic transmission from birds and swine, but additional mutations are required to allow for those viruses to transmit between humans (Cox and Subbarao, 2000; Harris et al., 2017).

The host tropism of the influenza virus is determined by the sialic acid species and its linkage to the underlying glycan. Most genomes of members of the deuterostomes contain a gene encoding for CMP-Neu5Ac hydroxylase (CMAH), the enzyme responsible for converting Neu5Ac to Neu5Gc (Peri et al., 2018). Deletions in CMAH have been described in humans (Chou et al., 1998), platypus (Schauer et al., 2009), ferrets (Ng et al., 2014), and New world monkeys (Springer et al., 2014), preventing the endogenous production of Neu5Gc, instead allowing decoration of glycans with Neu5Ac. All neuraminidases isolated from influenza viruses since year 1967 cleave both, α2,3- and α2,6-sialic acids (Baum and Paulson, 1990; Kobasa et al., 1999; Franca de Barros et al., 2003), with the neuraminidases of H1N1 and H3N2 cleaving α2,3-sialic acid more efficiently (Ng et al., 2014). While epithelial cells in the respiratory tract and intestine of birds, and tracheal cells of horses, mainly carry glycoconjugates having α2,3-linked sialic acids, the human trachea mainly contains cells carrying glycans with α2,6-linked sialic acids. Tracheal cells in the pigs express α2,6-linked and α2,3-linked sialic acids (Baum and Paulson, 1990; Ito et al., 1998). Accordingly, HA of avian and equine influenza viruses preferentially bind to α2,3-linked sialic acids, while HA of human influenza viruses has a higher affinity towards α2,6-linked sialic acids and swine influenza viruses bind both, α2,6-linked and α2,3-linked sialic acids (Krizanova and Rathova, 1969; Rogers and Paulson, 1983; Couceiro et al., 1993; Suzuki, 2005). Despite having different affinities towards the Sia-linkage to galactose, IAV is also influenced by the Sia species, although the specificity varies greatly among isolates.

Most IAV neuraminidases scavenge Neu5Ac and Neu5Gc from glycoconjugates, but have a lower efficiency for Neu5Gc (Xu et al., 1995; Broszeit et al., 2019; Barnard et al., 2020). Exceptions are NAs of H1N1 or viruses isolated between 1967 and 1969, which prefer Neu5Gc- over Neu5Ac-containing substrates (Kobasa et al., 1999; Ng et al., 2014). The interplay and balance in the specificities of the IAV hemagglutinin and neuraminidase is needed for successful viral infection (Guo et al., 2018; Broszeit et al., 2019). However, not only the sialic acid species and linkage determine IAV binding, but also modifications of other underlying carbohydrates of the glycan strand, like fucosylation, sulfation or phosphorylation of non-sialylated glycans affect IAV binding (Stevens et al., 2006; Byrd-Leotis et al., 2019). O-acetyl modification can inhibit HA binding and neuraminidase activity (Zimmer et al., 1994; Schauer, 2004; Barnard et al., 2020), but it is required for infection by other viruses like human coronaviruses OC43 and HKU1, as well as influenza C and D virus (Hulswit et al., 2019).

Interestingly, α2-6 sialylated glycans, expressed on the epithelial cells of the upper respiratory tract in humans attract seasonal influenza viruses, with inflammation limited to this location and usually milder disease. The highly pathogenic avian H5N1 influenza virus mainly binds to α2-3 sialylated glycans and primarily infects type 2 pneumocytes in the human lung, often leading to severe pneumonia (Shinya et al., 2006). Due to mutations in the HA, H5N1 viruses can bind both α2-3 and α2-6 sialylated glycans (Yamada et al., 2006), making it easier for the virus to spread from human to human. The H1N1 2009 virus is special as it acquired a D222G substitution in HA, detected in severe and fatal cases, which changes the receptor binding specificity from α2-6 to α2-3 sialylated glycans and allows the virus to infect ciliated bronchial cells, possibly increasing the severity of pneumonia (Liu et al., 2010; Mak et al., 2010).

Other viruses that predispose the host for secondary bacterial infections include respiratory syncytial virus (RSV), rhinovirus (RV), human coronavirus, parainfluenza virus and adenovirus (AV) (Falsey et al., 2013). Whereas parainfluenza virus also uses host sialic acids to attach to cells, syncytial virus, rhinovirus, parainfluenza virus and adenovirus utilize diverse attachment receptors (Moscona, 2005; Bochkov and Gern, 2016; Battles and McLellan, 2019; Stasiak and Stehle, 2020). The novel coronavirus SARS-CoV-2 binds the cellular receptor Angiotensin-converting enzyme 2 (ACE2) to cause coronavirus disease 2019 (COVID-19) (Yan et al., 2020). SARS-CoV-2 may be better in causing lung infection due to its greater binding affinity for the ACE2 receptors, which are present on epithelial cells in the lower airways. ACE2 receptors are also expressed on endothelial cells, allowing the virus to cause thrombosis and other vascular effects that greatly contribute to morbidity in COVID-19 patients (Ackermann et al., 2020; Cure and Cure, 2020; Sardu et al., 2020).

The mechanisms driving viral bacterial co-pathogenesis are diverse and complex, but often similar for the different viruses, including damage of the airways and dysregulated immune responses which, in turn, supports bacterial growth, adherence and invasion into normally sterile body sites. Similarities between influenza- and SARS-CoV-2-mediated host immune responses in severely sick patients that might favor bacterial coinfection include the damaged lung epithelium and the hyperactive immune response with increased levels of cytokines and pulmonary infiltration of immune cells (de Jong et al., 2006; Kash et al., 2006; Perrone et al., 2008; Huang et al., 2020; Lucas et al., 2020; Karki et al., 2021). Until now, we know little regarding potential bacterial, especially, pneumococcal coinfections, in COVID-19 patients. Frequencies of coinfections in COVID-19 patients range from 3.5% for confirmed community-onset bacterial infection (Vaughn et al., 2020) to 28% in severely ill patients from intensive care units (ICUs) (Contou et al., 2020; Feng et al., 2020; Zhou et al., 2020). In one study secondary bacterial infection, defined as a positive blood or LRT culture, occurred in 15% of all patients with 50% frequency in non-survivors compared to only 1% in survivors (Zhou et al., 2020). Another study, using throat swab samples and PCR, identified 24 different respiratory pathogens of which *S. pneumoniae* was the most common, followed by *Klebsiella pneumoniae* and *Haemophilus influenzae* (Zhu et al., 2020). A recent review summarized that only 1.3% of 522 patients in ICUs developed nosocomial superinfections with antimicrobial resistant bacteria, suggesting that COVID-19 overall associates less with bacterial infections, and the isolated bacterial pathogens differ from those causing lower respiratory tract infections during influenza pandemics, with *S. pneumoniae* isolated rarely (Fattorini et al., 2020). However, the methods and definitions used to identify bacterial coinfections are diverse, and the role of coinfection on clinical course and outcomes of COVID-19 has not been investigated yet. A recent summary demonstrates that about 16% of hospitalized COVID-19 patients develop secondary bacterial infection (Rawson et al., 2020) which requires antibiotic therapy. Another study analyzing the antibiotic use in patients with COVID-19 revealed that the prevalence of antibiotic prescribing was around 75% and it was higher with increasing patient age and with increasing proportion of patients requiring mechanical ventilation (Langford et al., 2021). In general, antimicrobial resistance is increasing worldwide (Klein et al., 2021) and a major driver is overuse and misuse of antibiotics. Thus, the ongoing pandemic of antimicrobial resistance may further increase, urging us to develop new strategies that help to prevent and treat viral-bacterial coinfection.

## Current Treatment Approaches for Secondary Pneumococcal Pneumonia

In general, prevention may be easier than cure, and vaccines against both influenza and pneumococci can reduce the coinfection aspect. Vaccines against influenza have been shown to reduce both the viral infection and associated secondary pneumococcal infections in mice (Huber et al., 2010; Sun et al., 2011; Haynes et al., 2012; Mina et al., 2013). However, the strong adaptive immune response evoked by viral vaccination compromises innate antibacterial defenses similar to what is observed for the viral infection itself. Indeed, vaccination of mice with live attenuated influenza virus primes the upper respiratory tract for increased bacterial colonization and promotes pneumococcal transmigration to other body sites as seen following influenza virus infection (Mina et al., 2014; Mina et al., 2015), but it can prevent invasive bacterial disease (Sun et al., 2011). In humans, presence of virus is associated with increased pneumococcal carriage (Glennie et al., 2016), and the symptoms humans experience during live viral vaccination are linked to nasal colonization with pneumococci (Hales et al., 2020), suggesting that the immunological changes occurring as a result of host-microbial interactions in the upper respiratory tract might allow aspiration of the bacteria and thus promote infection in the lower airways. Studies in both humans and mice agree that initial contact with influenza (or live-attenuated vaccine) increases the susceptibility to *Streptococcus pneumoniae* infection (de Steenhuijsen Piters et al., 2019; Rylance et al., 2019).

Pneumococcal conjugate vaccines are successful in reducing the overall incidence of invasive pneumococcal disease (IPD) in vaccinated children (Klugman, 2001), and reduce severe influenza-pneumococcal coinfections of the LRT in vaccinated individuals (Madhi et al., 2004). However, vaccines against pneumococci have limited efficiency in other older age groups due to the emergence of non-vaccine type pneumococcal strains in IPD and carriage. Thus, their effectiveness in reducing co-infections between influenza and pneumococci might be limited, and in mice and humans pneumococcal conjugate vaccine have been shown to protect only about 50% of the vaccinated individuals against secondary pneumococcal infection (Madhi et al., 2004; Mina et al., 2013; Metzger et al., 2015). Even though vaccines might be useful to reduce secondary pneumococcal infections, we have to bear in mind that unintended consequences can appear. Additionally, vaccines are not available against other bacteria that commonly cause secondary bacterial infection and the influenza virus is not the only virus predisposing the host for secondary bacterial infection. Thus, these strategies may only influence parts of the problem.

Besides vaccination, antiviral agents that repress viral replication like neuraminidase inhibitors such as Zanamivir and Oseltamivir effectively inhibit disease progression and reduce influenza-related symptoms (Von Itzstein et al., 1993; Gubareva et al., 2000). However, this treatment does not reduce the viral load and has limited effects when administered later during infection (McCullers and Bartmess, 2003; McCullers, 2004). Despite the limited antiviral effect when treatment is given later during the course of the infection, delayed therapy until up to 5 days post infection improves survival, but does not completely prevent mortality in a mouse model of secondary pneumococcal pneumonia (McCullers, 2004). The underlying mechanism is unclear, but can possibly be explained by an antiviral effect on increased viral loads post-bacterial infection as detected during secondary bacterial infection (Smith et al., 2013). Fludase, a recombinant sialidase that prevents viral entry into epithelial cells by cleaving sialic acids (Malakhov et al., 2006), was suggested to reduce the risk of secondary pneumococcal infection in mice (Hedlund et al., 2010). Surprisingly, Fludase treatment 3 days prior to bacterial infection did not alter bacterial numbers, despite the ability of pneumococci to feed on free sialic acids (Hedlund et al., 2010; Siegel et al., 2014). Also treatment with neutralizing influenza antibodies reduces the disease severity with lower viral and bacterial numbers and reduced lung injury in a mouse model of secondary pneumococcal infection (Van Someren Greve et al., 2018). However, in both studies the effects of repetitive treatment, and treatment at later time points and/or during bacterial infection remain to be determined.

Antimicrobial agents also reduce disease severity and occurrence of secondary bacterial pneumonia (McCullers, 2004; Karlstrom et al., 2009; Karlstrom et al., 2011; Ghoneim et al., 2013), but treatment may be insufficient in improving mortality (Karlstrom et al., 2009), and antibiotic resistant bacteria may further complicate the use of antibiotics. Phage therapies may provide a valuable alternative to antibiotics for treating secondary bacterial infections, but its efficacy in virus-infected patients must be evaluated in clinical studies (Manohar et al., 2020).

Immunomodulatory therapies, like treatment with IFNs or IFN antagonist, have been suggested earlier, but seem to induce more complex effects on the immune response than previously expected (Davidson et al., 2015; Metzger et al., 2015). The inflammation-induced leakage that does not only lead to acute respiratory distress syndrome, but also provides nutrient for bacteria to feed on (Sender et al., 2020), can be at least partly prevented by treatment with soluble ligands that reduce vascular permeability in the lungs and other organs and improve survival in animal models (London et al., 2010). Systemic administration of antioxidants as immunomodulatory therapy to neutralize virus-induced oxidative stress and increase macrophage activity improves survival in influenza-pneumococci coinfected mice (Wu et al., 2017; Gonzalez-Juarbe et al., 2020). However, antioxidant treatment may affect pneumococcal growth, as observed in our study (Sender et al., 2020), but whether route, dose and time of antioxidant administration may affect disease outcome differently remains to be determined. A recent study suggests a dual-functioning broad-spectrum virus- and host-targeting peptide against respiratory viruses, including influenza virus and SARS-CoV-2, as a promising candidate to prevent viral infection (Zhao et al., 2020). Even though an encouraging approach, where one compound combines two targets (virus and host), has been suggested, further studies are needed to elucidate its impact on secondary bacterial infection.

In general, more detailed knowledge is needed on how infection processes change over time and the interaction between involved pathogens and host factors in order to improve our ability to develop new therapeutic strategies and/or targets that effectively abrogate and/or cure secondary bacterial infection. A recently evolving research avenue is to target specific bacterial factors. In that regard, targeting pneumococcal surface protein A (PspA), a major surface protein of pneumococci and a promising vaccine target, might be an interesting approach to evoke protective antibody responses and promotion of bacterial clearance during secondary bacterial infection, (King et al., 2009; Kong et al., 2013; Greene et al., 2016), but the effect depends on the infectious pneumococcal dose (Roberts et al., 2019). The limited protection of currently available therapies, including their time-dependent efficacy and possible adverse effects, in addition to the growing problem of antibiotic resistance, underlines the need of new preventative and therapeutic strategies. Kinetic models can help us to determine the efficacy needed for successful treatment, identify potential immune effects, and show how the regulation of underlying mechanisms can be used to design new therapeutic strategies (Smith, 2017). An attractive alternative approach to improve treatment success or even prevent secondary bacterial infection could be to combine targeted antibacterial therapy with antiviral and/or immunomodulatory therapy. Conflicting results and the problem with extrapolating results from animal models to human therapy should be considered in the attempts to identify and implement novel more specific and effective treatments.

## Concluding Remarks

Worldwide, LRTI and pneumonia is the leading cause of morbidity and mortality, accounting for more than 4 million deaths yearly (World Health Organization, 2017). A systematic analysis of the global burden of LRTI estimated that these diseases caused about 2.4 million deaths in 2016, of which almost 1.2 million deaths were attributed to the pneumococcus, the leading cause of both morbidity and mortality among LRTIs (GBD 2016 Lower Respiratory Infections Collaborators, 2018). One of the major risk factors for the development of severe pneumococcal disease is preceding viral infections, especially with influenza A virus. LRTIs linked to influenza caused about 145,000 deaths worldwide in 2017, according to an analysis from the Global Burden of Disease Study (GBD 2017 Influenza Collaborators, 2019). Fatality from influenza is often linked to secondary bacterial infections. The mechanisms driving virulent coinfections are complex and replete, including dysregulated lung physiology, with impaired mucociliary clearance, and modulation of host immune responses caused by the virus, which in turn promotes bacterial growth, adherence and invasion into normally sterile sites of the lungs. Recently evolving research focuses on the role of specific bacterial factors and investigates how pneumococci sense and adapt to virus-induced changes in the environment. The currently ongoing COVID-19 pandemic has already caused more than 2.3 million deaths worldwide (World Health Organization, 2020) with numbers increasing. Our current knowledge regarding secondary bacterial infections in COVID-19 patients is still limited, but considering that both influenza and SARS-CoV-2 cause similar disease symptoms with a massive inflammatory immune response in the lower respiratory tract, ultimately leading to acute respiratory distress syndrome, a predisposition for bacterial superinfections is likely. The prophylactic use of antibiotics has increased due to the currently ongoing SARS-Cov-2 pandemic, enhancing the risk for increasing resistance to antibiotics. A better understanding of the mechanisms that promote bacterial superinfection, and more knowledge regarding the processes and factors bacteria use to successfully establish disease in virally infected environments, will help us to develop new therapeutic strategies and identify targets that effectively abrogate and/or cure secondary bacterial infections.

## Author Contributions

VS, KH, and BH-N all contributed to the design, analysis, and collection of data, as well as to write the manuscript. All authors contributed to the article and approved the submitted version.

## Funding

Fundings were provided by Knut and Alice Wallenberg foundation, the Swedish Research Council, and Stockholm County Council.

## Conflict of Interest

The authors declare that the research was conducted in the absence of any commercial or financial relationships that could be construed as a potential conflict of interest.

## References

[B1] Abramson J. S. Mills E. L. Giebink G. S. Quie P. G. (1982). Depression of monocyte and polymorphonuclear leukocyte oxidative metabolism and bactericidal capacity by influenza A virus. Infect. Immun. 35, 350–355. 10.1128/IAI.35.1.350-355.1982 7054126 PMC351036

[B2] Ackermann M. Verleden S. E. Kuehnel M. Haverich A. Welte T. Laenger F.. (2020). Pulmonary Vascular Endothelialitis, Thrombosis, and Angiogenesis in Covid-19. N. Engl. J. Med. 383, 120–128. 10.1056/NEJMoa2015432 32437596 PMC7412750

[B3] Akhter F. Womack E. Vidal J. E. Le Breton Y. Mciver K. S. Pawar S.. (2020). Hemoglobin stimulates vigorous growth of Streptococcus pneumoniae and shapes the pathogen’s global transcriptome. Sci. Rep. 10, 15202. 10.1038/s41598-020-71910-1 32938947 PMC7494912

[B4] Arredouani M. Yang Z. Ning Y. Qin G. Soininen R. Tryggvason K.. (2004). The scavenger receptor MARCO is required for lung defense against pneumococcal pneumonia and inhaled particles. J. Exp. Med. 200, 267–272. 10.1084/jem.20040731 15263032 PMC2212010

[B5] Astry C. L. Jakab G. J. (1984). Influenza virus-induced immune complexes suppress alveolar macrophage phagocytosis. J. Virol. 50, 287–292. 10.1128/JVI.50.2.287-292.1984 6708169 PMC255619

[B6] Barnard K. N. Alford-Lawrence B. K. Buchholz D. W. Wasik B. R. Laclair J. R. Yu H.. (2020). Modified Sialic Acids on Mucus and Erythrocytes Inhibit Influenza A Virus Hemagglutinin and Neuraminidase Functions. J. Virol. 94 (9), e01567–19. 10.1128/JVI.01567-19 32051275 PMC7163148

[B7] Battles M. B. Mclellan J. S. (2019). Respiratory syncytial virus entry and how to block it. Nat. Rev. Microbiol. 17, 233–245. 10.1038/s41579-019-0149-x 30723301 PMC7096974

[B8] Baum L. G. Paulson J. C. (1990). Sialyloligosaccharides of the respiratory epithelium in the selection of human influenza virus receptor specificity. Acta Histochem. Suppl. 40, 35–38. 2091044

[B9] Bengoechea J. A. Bamford C. G. (2020). SARS-CoV-2, bacterial co-infections, and AMR: the deadly trio in COVID-19? EMBO Mol. Med. 12, e12560. 10.15252/emmm.202012560 32453917 PMC7283846

[B10] Bochkov Y. A. Gern J. E. (2016). Rhinoviruses and Their Receptors: Implications for Allergic Disease. Curr. Allergy Asthma Rep. 16, 30. 10.1007/s11882-016-0608-7 26960297 PMC4854667

[B11] Bogaert D. De Groot R. Hermans P. W. (2004). Streptococcus pneumoniae colonisation: the key to pneumococcal disease. Lancet Infect. Dis. 4, 144–154. 10.1016/S1473-3099(04)00938-7 14998500

[B12] Broszeit F. Tzarum N. Zhu X. Nemanichvili N. Eggink D. Leenders T.. (2019). N-Glycolylneuraminic Acid as a Receptor for Influenza A Viruses. Cell Rep. 3284-3294, e3286. 10.1016/j.celrep.2019.05.048 PMC6750725 31189111

[B13] Buckwalter C. M. King S. J. (2012). Pneumococcal carbohydrate transport: food for thought. Trends Microbiol. 20, 517–522. 10.1016/j.tim.2012.08.008 22959614 PMC4630977

[B14] Burnaugh A. M. Frantz L. J. King S. J. (2008). Growth of Streptococcus pneumoniae on human glycoconjugates is dependent upon the sequential activity of bacterial exoglycosidases. J. Bacteriol. 190, 221–230. 10.1128/JB.01251-07 17981977 PMC2223752

[B15] Byrd-Leotis L. Jia N. Dutta S. Trost J. F. Gao C. Cummings S. F.. (2019). Influenza binds phosphorylated glycans from human lung. Sci. Adv. 5, eaav2554. 10.1126/sciadv.aav2554 30788437 PMC6374103

[B16] Canton R. Gijon D. Ruiz-Garbajosa P. (2020). Antimicrobial resistance in ICUs: an update in the light of the COVID-19 pandemic. Curr. Opin. Crit. Care 26, 433–441. 10.1097/MCC.0000000000000755 32739970

[B17] Cao J. Wang D. Xu F. Gong Y. Wang H. Song Z.. (2014). Activation of IL-27 signalling promotes development of postinfluenza pneumococcal pneumonia. EMBO Mol. Med. 6, 120–140. 10.1002/emmm.201302890 24408967 PMC3936494

[B18] Cassone M. Gagne A. L. Spruce L. A. Seeholzer S. H. Sebert M. E. (2012). The HtrA protease from Streptococcus pneumoniae digests both denatured proteins and the competence-stimulating peptide. J. Biol. Chem. 287, 38449–38459. 10.1074/jbc.M112.391482 23012372 PMC3493890

[B19] Cawcutt K. Kalil A. C. (2017). Pneumonia with bacterial and viral coinfection. Curr. Opin. Crit. Care 23, 385–390. 10.1097/MCC.0000000000000435 28777158

[B20] Centers for Disease Control and Prevention (2009). Bacterial coinfections in lung tissue specimens from fatal cases of 2009 pandemic influenza A (H1N1) - United States, May-August 2009. Available at: https://www.cdc.gov/mmwr/preview/mmwrhtml/mm5838a4.htm (Accessed Feb 03 2021). 19798021

[B21] Chen W. H. Toapanta F. R. Shirey K. A. Zhang L. Giannelou A. Page C.. (2012). Potential role for alternatively activated macrophages in the secondary bacterial infection during recovery from influenza. Immunol. Lett. 141, 227–234. 10.1016/j.imlet.2011.10.009 22037624 PMC3243824

[B22] Chertow D. S. Memoli M. J. (2013). Bacterial coinfection in influenza: a grand rounds review. JAMA 309, 275–282. 10.1001/jama.2012.194139 23321766

[B23] Chockalingam A. K. Hickman D. Pena L. Ye J. Ferrero A. Echenique J. R.. (2012). Deletions in the neuraminidase stalk region of H2N2 and H9N2 avian influenza virus subtypes do not affect postinfluenza secondary bacterial pneumonia. J. Virol. 86, 3564–3573. 10.1128/JVI.05809-11 22278240 PMC3302490

[B24] Chou H. H. Takematsu H. Diaz S. Iber J. Nickerson E. Wright K. L.. (1998). A mutation in human CMP-sialic acid hydroxylase occurred after the Homo-Pan divergence. Proc. Natl. Acad. Sci. U. S. A. 95, 11751–6. 10.1073/pnas.95.20.11751 PMC21712 9751737

[B25] Contou D. Claudinon A. Pajot O. Micaelo M. Longuet Flandre P. Dubert M.. (2020). Bacterial and viral co-infections in patients with severe SARS-CoV-2 pneumonia admitted to a French ICU. Ann. Intensive Care 10, 119. 10.1186/s13613-020-00736-x 32894364 PMC7475952

[B26] Couceiro J. N. Paulson J. C. Baum L. G. (1993). Influenza virus strains selectively recognize sialyloligosaccharides on human respiratory epithelium; the role of the host cell in selection of hemagglutinin receptor specificity. Virus Res. 29, 155–165. 10.1016/0168-1702(93)90056-S 8212857

[B27] Cox N. J. Subbarao K. (2000). Global epidemiology of influenza: past and present. Annu. Rev. Med. 51, 407–421. 10.1146/annurev.med.51.1.407 10774473

[B28] Cure E. Cure M. C. (2020). COVID-19 May Predispose to Thrombosis by Affecting Both Vascular Endothelium and Platelets. Clin. Appl. Thromb. Hemost. 26, 1076029620933945. 10.1177/1076029620933945 32619104 PMC7495934

[B29] Davidson S. Maini M. K. Wack A. (2015). Disease-promoting effects of type I interferons in viral, bacterial, and coinfections. J. Interferon Cytokine Res. 35, 252–264. 10.1089/jir.2014.0227 25714109 PMC4389918

[B30] De Jong M. D. Simmons C. P. Thanh T. T. Hien V. M. Smith G. J. Chau T. N.. (2006). Fatal outcome of human influenza A (H5N1) is associated with high viral load and hypercytokinemia. Nat. Med. 12, 1203–1207. 10.1038/nm1477 16964257 PMC4333202

[B31] De Steenhuijsen Piters W. Jochems S. P. Mitsi E. Rylance J. Pojar S. Nikolaou E.. (2019). Interaction between the nasal microbiota and S. pneumoniae in the context of live-attenuated influenza vaccine. Nat. Commun. 10, 2981. 10.1038/s41467-019-10814-9 31278315 PMC6611866

[B32] Debets-Ossenkopp Y. Mills E. L. Van Dijk W. C. Verbrugh H. A. Verhoef J. (1982). Effect of influenza virus on phagocytic cells. Eur. J. Clin. Microbiol. 1, 171–177. 10.1007/BF02019619 7173182

[B33] Dela Cruz C. S. Liu W. He C. H. Jacoby A. Gornitzky A. Ma B.. (2012). Chitinase 3-like-1 promotes Streptococcus pneumoniae killing and augments host tolerance to lung antibacterial responses. Cell Host Microbe 12, 34–46. 10.1016/j.chom.2012.05.017 22817986 PMC3613130

[B34] Didierlaurent A. Goulding J. Patel S. Snelgrove R. Low L. Bebien M.. (2008). Sustained desensitization to bacterial Toll-like receptor ligands after resolution of respiratory influenza infection. J. Exp. Med. 205, 323–329. 10.1084/jem.20070891 18227219 PMC2271005

[B35] Dona D. Di Chiara C. Sharland M. (2020). Multi-drug-resistant infections in the COVID-19 era: a framework for considering the potential impact. J. Hosp. Infect. 106, 198–199. 10.1016/j.jhin.2020.05.020 32425287 PMC7231493

[B36] Ellis G. T. Davidson S. Crotta S. Branzk N. Papayannopoulos V. Wack A. (2015). TRAIL+ monocytes and monocyte-related cells cause lung damage and thereby increase susceptibility to influenza-Streptococcus pneumoniae coinfection. EMBO Rep. 16, 1203–1218. 10.15252/embr.201540473 26265006 PMC4576987

[B37] Falsey A. R. Becker K. L. Swinburne A. J. Nylen E. S. Formica M. A. Hennessey P. A.. (2013). Bacterial complications of respiratory tract viral illness: a comprehensive evaluation. J. Infect. Dis. 208, 432–441. 10.1093/infdis/jit190 23661797 PMC3699009

[B38] Fattorini L. Creti R. Palma C. Pantosti A. Unit of Antibiotic, R., Special, P., Unit of Antibiotic, R., and Special Pathogens of the Department of Infectious Diseases, I.S.D.S.R. (2020). Bacterial coinfections in COVID-19: an underestimated adversary. Ann. Ist. Super. Sanita 56, 359–364. 10.4415/ANN_20_03_14 32959802

[B39] Feng Y. Ling Y. Bai T. Xie Y. Huang J. Li J.. (2020). COVID-19 with Different Severities: A Multicenter Study of Clinical Features. Am. J. Respir. Crit. Care Med. 201, 1380–1388. 10.1164/rccm.202002-0445OC 32275452 PMC7258639

[B40] Franca De Barros J. Jr. Sales Alviano D. Da Silva M. H. Dutra Wigg M. Sales Alviano C. Schauer R.. (2003). Characterization of sialidase from an influenza A (H3N2) virus strain: kinetic parameters and substrate specificity. Intervirology 46, 199–206. 10.1159/000072428 12931027

[B41] GBD 2016 Lower Respiratory Infections Collaborators (2018). Estimates of the global, regional, and national morbidity, mortality, and aetiologies of lower respiratory infections in 195 countries 1990-2016: a systematic analysis for the Global Burden of Disease Study 2016. Lancet Infect. Dis. 18, 1191–1210. 10.1016/S1473-3099(18)30310-4 30243584 PMC6202443

[B42] GBD 2017 Influenza Collaborators (2019). “Mortality, morbidity, and hospitalisations due to influenza lower respiratory tract infections 2017: an analysis for the Global Burden of Disease Study 2017”. Lancet Respir. Med. 7, 69–89. 10.1016/S2213-2600(18)30496-X PMC6302221 30553848

[B43] Getahun H. Smith I. Trivedi K. Paulin S. Balkhy H. H. (2020). Tackling antimicrobial resistance in the COVID-19 pandemic. Bull. World Health Organ 98, 442–442A. 10.2471/BLT.20.268573 32742026 PMC7375214

[B44] Ghaffar F. Friedland I. R. Mccracken G. H. Jr. (1999). Dynamics of nasopharyngeal colonization by Streptococcus pneumoniae. Pediatr. Infect. Dis. J. 18, 638–646. 10.1097/00006454-199907000-00016 10440444

[B45] Ghoneim H. E. Thomas P. G. McCullers J. A. (2013). Depletion of alveolar macrophages during influenza infection facilitates bacterial superinfections. J. Immunol. 191, 1250–1259. 10.4049/jimmunol.1300014 23804714 PMC4907362

[B46] Giamarellos-Bourboulis E. J. Raftogiannis M. Antonopoulou A. Baziaka F. Koutoukas P. Savva A.. (2009). Effect of the novel influenza A (H1N1) virus in the human immune system. PloS One 4, e8393. 10.1371/journal.pone.0008393 20037642 PMC2792719

[B47] Glennie S. Gritzfeld J. F. Pennington S. H. Garner-Jones M. Coombes N. Hopkins M. J.. (2016). Modulation of nasopharyngeal innate defenses by viral coinfection predisposes individuals to experimental pneumococcal carriage. Mucosal Immunol. 9, 56–67. 10.1038/mi.2015.35 25921341 PMC4703943

[B48] Gonzalez-Juarbe N. Gilley R. P. Hinojosa C. A. Bradley K. M. Kamei A. Gao G.. (2015). Pore-Forming Toxins Induce Macrophage Necroptosis during Acute Bacterial Pneumonia. PloS Pathog. 11, e1005337. 10.1371/journal.ppat.1005337 26659062 PMC4676650

[B49] Gonzalez-Juarbe N. Riegler A. N. Jureka A. S. Gilley R. P. Brand J. D. Trombley J. E.. (2020). Influenza-Induced Oxidative Stress Sensitizes Lung Cells to Bacterial-Toxin-Mediated Necroptosis. Cell Rep. 32, 108062. 10.1016/j.celrep.2020.108062 32846120 PMC7570217

[B50] Gottschalk A. (1958). The influenza virus neuraminidase. Nature 181, 377–378. 10.1038/181377a0 13504207

[B51] Gou X. Yuan J. Wang H. Wang X. Xiao J. Chen J.. (2019). IL-6 During Influenza-Streptococcus pneumoniae Co-Infected Pneumonia-A Protector. Front. Immunol. 10, 3102. 10.3389/fimmu.2019.03102 32038632 PMC6985362

[B52] Goulding J. Godlee A. Vekaria S. Hilty M. Snelgrove R. Hussell T. (2011). Lowering the threshold of lung innate immune cell activation alters susceptibility to secondary bacterial superinfection. J. Infect. Dis. 204, 1086–1094. 10.1093/infdis/jir467 21881124 PMC3164429

[B53] Greene C. J. Marks L. R. Hu J. C. Reddinger R. Mandell L. Roche-Hakansson H.. (2016). Novel Strategy To Protect against Influenza Virus-Induced Pneumococcal Disease without Interfering with Commensal Colonization. Infect. Immun. 84, 1693–1703. 10.1128/IAI.01478-15 27001538 PMC4907141

[B54] Guan W. J. Ni Z. Y. Hu Y. Liang W. H. Ou C. Q. He J. X.. (2020). Clinical Characteristics of Coronavirus Disease 2019 in China. N. Engl. J. Med. 382, 1708–1720. 10.1056/NEJMoa2002032 32109013 PMC7092819

[B55] Gubareva L. V. Kaiser L. Hayden F. G. (2000). Influenza virus neuraminidase inhibitors. Lancet 355, 827–835. 10.1016/S0140-6736(99)11433-8 10711940

[B56] Guo Z. Wilson J. R. York I. A. Stevens J. (2018). Biosensor-based epitope mapping of antibodies targeting the hemagglutinin and neuraminidase of influenza A virus. J. Immunol. Methods 461, 23–29. 10.1016/j.jim.2018.07.007 30053389 PMC6416777

[B57] Hales C. Jochems S. P. Robinson R. Solorzano C. Carniel B. Pojar S.. (2020). Symptoms associated with influenza vaccination and experimental human pneumococcal colonisation of the nasopharynx. Vaccine 38, 2298–2306. 10.1016/j.vaccine.2020.01.070 32035708 PMC7045083

[B58] Harris K. A. Freidl G. S. Munoz O. S. Von Dobschuetz S. De Nardi M. Wieland B.. (2017). Epidemiological Risk Factors for Animal Influenza A Viruses Overcoming Species Barriers. Ecohealth 14, 342–360. 10.1007/s10393-017-1244-y 28523412

[B59] Haynes L. Szaba F. M. Eaton S. M. Kummer L. W. Lanthier P. A. Petell A. H.. (2012). Immunity to the conserved influenza nucleoprotein reduces susceptibility to secondary bacterial infections. J. Immunol. 189, 4921–4929. 10.4049/jimmunol.1201916 23028058 PMC3490014

[B60] Hedlund M. Aschenbrenner L. M. Jensen K. Larson J. L. Fang F. (2010). Sialidase-based anti-influenza virus therapy protects against secondary pneumococcal infection. J. Infect. Dis. 201, 1007–1015. 10.1086/651170 20170378 PMC2874251

[B61] Hentrich K. Lofling J. Pathak A. Nizet V. Varki A. Henriques-Normark B. (2016). Streptococcus pneumoniae Senses a Human-like Sialic Acid Profile via the Response Regulator CiaR. Cell Host Microbe 20, 307–317. 10.1016/j.chom.2016.07.019 27593514 PMC5025396

[B62] Hjalmarsdottir M. A. Gumundsdottir P. F. Erlendsdottir H. Kristinsson K. G. Haraldsson G. (2016). Cocolonization of Pneumococcal Serotypes in Healthy Children Attending Day Care Centers: Molecular Versus Conventional Methods. Pediatr. Infect. Dis. J. 35, 477–480. 10.1097/INF.0000000000001059 26808723

[B63] Huang C. Wang Y. Li X. Ren L. Zhao J. Hu Y.. (2020). Clinical features of patients infected with 2019 novel coronavirus in Wuhan, China. Lancet 395, 497–506. 10.1016/S0140-6736(20)30183-5 31986264 PMC7159299

[B64] Huber V. C. Peltola V. Iverson A. R. McCullers J. A. (2010). Contribution of vaccine-induced immunity toward either the HA or the NA component of influenza viruses limits secondary bacterial complications. J. Virol. 84, 4105–4108. 10.1128/JVI.02621-09 20130054 PMC2849504

[B65] Hulswit R. J. G. Lang Y. Bakkers M. J. G. Li W. Li Z. Schouten A.. (2019). Human coronaviruses OC43 and HKU1 bind to 9-O-acetylated sialic acids via a conserved receptor-binding site in spike protein domain A. Proc. Natl. Acad. Sci. U. S. A. 116, 2681–2690. 10.1073/pnas.1809667116 30679277 PMC6377473

[B66] Hussell T. Cavanagh M. M. (2009). The innate immune rheostat: influence on lung inflammatory disease and secondary bacterial pneumonia. Biochem. Soc. Trans. 37, 811–813. 10.1042/BST0370811 19614599

[B67] Ito T. Couceiro J. N. Kelm S. Baum L. G. Krauss S. Castrucci M. R.. (1998). Molecular basis for the generation in pigs of influenza A viruses with pandemic potential. J. Virol. 72, 7367–7373. 10.1128/JVI.72.9.7367-7373.1998 9696833 PMC109961

[B68] Ivanov S. Renneson J. Fontaine J. Barthelemy A. Paget C. Fernandez E. M.. (2013). Interleukin-22 reduces lung inflammation during influenza A virus infection and protects against secondary bacterial infection. J. Virol. 87, 6911–6924. 10.1128/JVI.02943-12 23596287 PMC3676141

[B69] Karki R. Sharma B. R. Tuladhar S. Williams E. P. Zalduondo L. Samir P.. (2021). Synergism of TNF-alpha and IFN-gamma Triggers Inflammatory Cell Death, Tissue Damage, and Mortality in SARS-CoV-2 Infection and Cytokine Shock Syndromes. Cell 184 149-168, e117. 10.1101/2020.10.29.361048 PMC7674074 33278357

[B70] Karlstrom A. Boyd K. L. English B. K. McCullers J. A. (2009). Treatment with protein synthesis inhibitors improves outcomes of secondary bacterial pneumonia after influenza. J. Infect. Dis. 199, 311–319. 10.1086/596051 19113989 PMC2687083

[B71] Karlstrom A. Heston S. M. Boyd K. L. Tuomanen E. I. McCullers J. A. (2011). Toll-like receptor 2 mediates fatal immunopathology in mice during treatment of secondary pneumococcal pneumonia following influenza. J. Infect. Dis. 204, 1358–1366. 10.1093/infdis/jir522 21900488 PMC3218647

[B72] Karwelat D. Schmeck B. Ringel M. Benedikter B. J. Hubner K. Beinborn I.. (2020). Influenza virus-mediated suppression of bronchial Chitinase-3-like 1 secretion promotes secondary pneumococcal infection. FASEB J. 34, 16432–16448. 10.1096/fj.201902988RR 33095949

[B73] Kash J. C. Tumpey T. M. Proll S. C. Carter V. Perwitasari O. Thomas M. J.. (2006). Genomic analysis of increased host immune and cell death responses induced by 1918 influenza virus. Nature 443, 578–581. 10.1038/nature05181 17006449 PMC2615558

[B74] Kash J. C. Walters K. A. Davis A. S. Sandouk A. Schwartzman L. M. Jagger B. W.. (2011). Lethal synergism of 2009 pandemic H1N1 influenza virus and Streptococcus pneumoniae coinfection is associated with loss of murine lung repair responses. mBio 2 (5), e00172-11. 10.1128/mBio.00172-11 21933918 PMC3175626

[B75] King Q. O. Lei B. Harmsen A. G. (2009). Pneumococcal surface protein A contributes to secondary Streptococcus pneumoniae infection after influenza virus infection. J. Infect. Dis. 200, 537–545. 10.1086/600871 19586418 PMC2735857

[B76] Klein E. Y. Milkowska-Shibata M. Tseng K. K. Sharland M. Gandra S. Pulcini C.. (2021). Assessment of WHO antibiotic consumption and access targets in 76 countries 2000-15: an analysis of pharmaceutical sales data. Lancet Infect. Dis. 21, 107–115. 10.1016/S1473-3099(20)30332-7 32717205

[B77] Klugman K. P. (2001). Efficacy of pneumococcal conjugate vaccines and their effect on carriage and antimicrobial resistance. Lancet Infect. Dis. 1, 85–91. 10.1016/S1473-3099(01)00063-9 11871480

[B78] Kobasa D. Kodihalli S. Luo M. Castrucci M. R. Donatelli I. Suzuki Y.. (1999). Amino acid residues contributing to the substrate specificity of the influenza A virus neuraminidase. J. Virol. 73, 6743–6751. 10.1128/JVI.73.8.6743-6751.1999 10400772 PMC112759

[B79] Kong I. G. Sato A. Yuki Y. Nochi T. Takahashi H. Sawada S.. (2013). Nanogel-based PspA intranasal vaccine prevents invasive disease and nasal colonization by Streptococcus pneumoniae. Infect. Immun. 81, 1625–1634. 10.1128/IAI.00240-13 23460513 PMC3647999

[B80] Krizanova O. Rathova V. (1969). Serum inhibitors of myxoviruses. Curr. Top. Microbiol. Immunol. 47, 125–151. 10.1007/978-3-642-46160-6_6 4305308

[B81] Kuri T. Sorensen A. S. Thomas S. Karlsson Hedestam G. B. Normark S. Henriques-Normark B.. (2013). Influenza A virus-mediated priming enhances cytokine secretion by human dendritic cells infected with Streptococcus pneumoniae. Cell Microbiol. 15, 1385–1400. 10.1111/cmi.12122 23421931 PMC3798092

[B82] Langford B. J. So M. Raybardhan S. Leung V. Soucy J. R. Westwood D.. (2021). Antibiotic prescribing in patients with COVID-19: rapid review and meta-analysis. Clin. Microbiol. Infect. S1198–743X(20), E6.30778–3. 10.1016/j.cmi.2020.12.018 PMC7785281 33418017

[B83] Leekha S. Terrell C. L. Edson R. S. (2011). General principles of antimicrobial therapy. Mayo Clin. Proc. 86, 156–167. 10.4065/mcp.2010.0639 21282489 PMC3031442

[B84] Lemessurier K. S. Ogunniyi A. D. Paton J. C. (2006). Differential expression of key pneumococcal virulence genes in vivo. Microbiol. (Reading) 152, 305–311. 10.1099/mic.0.28438-0 16436418

[B85] Levine A. M. Koeningsknecht V. Stark J. M. (2001). Decreased pulmonary clearance of S. pneumoniae following influenza A infection in mice. J. Virol. Methods 94, 173–186. 10.1016/S0166-0934(01)00287-7 11337052

[B86] Li W. Moltedo B. Moran T. M. (2012). Type I interferon induction during influenza virus infection increases susceptibility to secondary Streptococcus pneumoniae infection by negative regulation of gammadelta T cells. J. Virol. 86, 12304–12312. 10.1128/JVI.01269-12 22951826 PMC3486468

[B87] Lindstrand A. Galanis I. Darenberg J. Morfeldt E. Naucler P. Blennow M.. (2016). Unaltered pneumococcal carriage prevalence due to expansion of non-vaccine types of low invasive potential 8years after vaccine introduction in Stockholm, Sweden. Vaccine 34, 4565–4571. 10.1016/j.vaccine.2016.07.031 27473304

[B88] Liu W. Tang F. Li Z. D. Yang H. Cao W. C. (2010). Characteristics derived from outbreaks of pandemic influenza A (H1N1) 2009 virus. Clin. Infect. Dis. 50, 622–623. 10.1086/650179 20095842

[B89] Liu X. Kimmey J. M. Matarazzo L. De Bakker V. Van Maele L. Sirard J. C.. (2020). Exploration of Bacterial Bottlenecks and Streptococcus pneumoniae Pathogenesis by CRISPRi-Seq. Cell Host Microbe. 29 (1), 107–120.E6. 10.1016/j.chom.2020.10.001 PMC7855995 33120116

[B90] London N. R. Zhu W. Bozza F. A. Smith M. C. Greif D. M. Sorensen L. K.. (2010). Targeting Robo4-dependent Slit signaling to survive the cytokine storm in sepsis and influenza. Sci. Transl. Med. 2, 23ra19. 10.1126/scitranslmed.3000678 PMC2875996 20375003

[B91] Lucas C. Wong P. Klein J. Castro T. B. R. Silva J. Sundaram M.. (2020). Longitudinal analyses reveal immunological misfiring in severe COVID-19. Nature 584, 463–469. 10.1038/s41586-020-2588-y 32717743 PMC7477538

[B92] Madhi S. A. Klugman K. P. Vaccine Trialist G. (2004). A role for Streptococcus pneumoniae in virus-associated pneumonia. Nat. Med. 10, 811–813. 10.1038/nm1077 15247911 PMC7095883

[B93] Mak G. C. Au K. W. Tai L. S. Chuang K. C. Cheng K. C. Shiu T. C.. (2010). Association of D222G substitution in haemagglutinin of 2009 pandemic influenza A (H1N1) with severe disease. Euro Surveill 15 (14), 19534. 10.2807/ese.15.14.19534-en 20394715

[B94] Malakhov M. P. Aschenbrenner L. M. Smee D. F. Wandersee M. K. Sidwell R. W. Gubareva L. V.. (2006). Sialidase fusion protein as a novel broad-spectrum inhibitor of influenza virus infection. Antimicrob. Agents Chemother. 50, 1470–1479. 10.1128/AAC.50.4.1470-1479.2006 16569867 PMC1426979

[B95] Manohar P. Loh B. Athira S. Nachimuthu R. Hua X. Welburn S. C.. (2020). Secondary Bacterial Infections During Pulmonary Viral Disease: Phage Therapeutics as Alternatives to Antibiotics? Front. Microbiol. 11, 1434. 10.3389/fmicb.2020.01434 32733404 PMC7358648

[B96] Marion C. Burnaugh A. M. Woodiga S. A. King S. J. (2011). Sialic acid transport contributes to pneumococcal colonization. Infect. Immun. 79, 1262–1269. 10.1128/IAI.00832-10 21189320 PMC3067482

[B97] McAuley J. L. Gilbertson B. P. Trifkovic S. Brown L. E. Mckimm-Breschkin J. L. (2019). Influenza Virus Neuraminidase Structure and Functions. Front. Microbiol. 10, 39. 10.3389/fmicb.2019.00039 30761095 PMC6362415

[B98] McCombs J. E. Kohler J. J. (2016). Pneumococcal Neuraminidase Substrates Identified through Comparative Proteomics Enabled by Chemoselective Labeling. Bioconjug Chem. 27, 1013–1022. 10.1021/acs.bioconjchem.6b00050 26954852 PMC4838540

[B99] McCullers J. A. Bartmess K. C. (2003). Role of neuraminidase in lethal synergism between influenza virus and Streptococcus pneumoniae. J. Infect. Dis. 187, 1000–1009. 10.1086/368163 12660947

[B100] McCullers J. A. Rehg J. E. (2002). Lethal synergism between influenza virus and Streptococcus pneumoniae: characterization of a mouse model and the role of platelet-activating factor receptor. J. Infect. Dis. 186, 341–350. 10.1086/341462 12134230

[B101] McCullers J. A. Mcauley J. L. Browall S. Iverson A. R. Boyd K. L. Henriques Normark B. (2010). Influenza enhances susceptibility to natural acquisition of and disease due to Streptococcus pneumoniae in ferrets. J. Infect. Dis. 202, 1287–1295. 10.1086/656333 20822454 PMC3249639

[B102] McCullers J. A. (2004). Effect of antiviral treatment on the outcome of secondary bacterial pneumonia after influenza. J. Infect. Dis. 190, 519–526. 10.1086/421525 15243927

[B103] McNamee L. A. Harmsen A. G. (2006). Both influenza-induced neutrophil dysfunction and neutrophil-independent mechanisms contribute to increased susceptibility to a secondary Streptococcus pneumoniae infection. Infect. Immun. 74, 6707–6721. 10.1128/IAI.00789-06 16982840 PMC1698099

[B104] Metzger D. W. Furuya Y. Salmon S. L. Roberts S. Sun K. (2015). Limited Efficacy of Antibacterial Vaccination Against Secondary Serotype 3 Pneumococcal Pneumonia Following Influenza Infection. J. Infect. Dis. 212, 445–452. 10.1093/infdis/jiv066 25649173 PMC4615793

[B105] Mina M. J. Klugman K. P. McCullers J. A. (2013). Live attenuated influenza vaccine, but not pneumococcal conjugate vaccine, protects against increased density and duration of pneumococcal carriage after influenza infection in pneumococcal colonized mice. J. Infect. Dis. 208, 1281–1285. 10.1093/infdis/jit317 23852122 PMC6281400

[B106] Mina M. J. McCullers J. A. Klugman K. P. (2014). Live attenuated influenza vaccine enhances colonization of Streptococcus pneumoniae and Staphylococcus aureus in mice. mBio 5 (1), e01040-13. 10.1128/mBio.01040-13 24549845 PMC3944816

[B107] Mina M. J. Klugman K. P. Rosch J. W. McCullers J. A. (2015). Live attenuated influenza virus increases pneumococcal translocation and persistence within the middle ear. J. Infect. Dis. 212, 195–201. 10.1093/infdis/jiu804 25505300 PMC4654764

[B108] Morens D. M. Taubenberger J. K. Fauci A. S. (2008). Predominant role of bacterial pneumonia as a cause of death in pandemic influenza: implications for pandemic influenza preparedness. J. Infect. Dis. 198, 962–970. 10.1086/591708 18710327 PMC2599911

[B109] Morris D. E. Cleary D. W. Clarke S. C. (2017). Secondary Bacterial Infections Associated with Influenza Pandemics. Front. Microbiol. 8, 1041. 10.3389/fmicb.2017.01041 28690590 PMC5481322

[B110] Moscona A. (2005). Entry of parainfluenza virus into cells as a target for interrupting childhood respiratory disease. J. Clin. Invest. 115, 1688–1698. 10.1172/JCI25669 16007245 PMC1159152

[B111] Muchmore E. A. Diaz S. Varki A. (1998). A structural difference between the cell surfaces of humans and the great apes. Am. J. Phys. Anthropol. 107, 187–198. 10.1002/(SICI)1096-8644(199810)107:2<187::AID-AJPA5>3.0.CO;2-S 9786333

[B112] Mucke P. A. Ostrzinski A. Hammerschmidt S. Maass S. Becher D. (2020). Proteomic Adaptation of Streptococcus pneumoniae to the Antimicrobial Peptide Human Beta Defensin 3 (hBD3) in Comparison to Other Cell Surface Stresses. Microorganisms 8 (11), 1697. 10.3390/microorganisms8111697 PMC7694020 33143252

[B113] Muller U. Steinhoff U. Reis L. F. Hemmi S. Pavlovic J. Zinkernagel R. M.. (1994). Functional role of type I and type II interferons in antiviral defense. Science 264, 1918–1921. 10.1126/science.8009221 8009221

[B114] Murray A. K. (2020). The Novel Coronavirus COVID-19 Outbreak: Global Implications for Antimicrobial Resistance. Front. Microbiol. 11, 1020. 10.3389/fmicb.2020.01020 32574253 PMC7237633

[B115] Nakamura S. Davis K. M. Weiser J. N. (2011). Synergistic stimulation of type I interferons during influenza virus coinfection promotes Streptococcus pneumoniae colonization in mice. J. Clin. Invest. 121, 3657–3665. 10.1172/JCI57762 21841308 PMC3163966

[B116] Ng P. S. Bohm R. Hartley-Tassell L. E. Steen J. A. Wang H. Lukowski S. W.. (2014). Ferrets exclusively synthesize Neu5Ac and express naturally humanized influenza A virus receptors. Nat. Commun. 5, 5750. 10.1038/ncomms6750 25517696 PMC4351649

[B117] Nogusa S. Thapa R. J. Dillon C. P. Liedmann S. Oguin T. H. 3rd Ingram J. P.. (2016). RIPK3 Activates Parallel Pathways of MLKL-Driven Necroptosis and FADD-Mediated Apoptosis to Protect against Influenza A Virus. Cell Host Microbe 20, 13–24. 10.1016/j.chom.2016.05.011 27321907 PMC5026823

[B118] Nugent K. M. Pesanti E. L. (1983). Tracheal function during influenza infections. Infect. Immun. 42, 1102–1108. 10.1128/IAI.42.3.1102-1108.1983 6642660 PMC264413

[B119] Parker R. B. Mccombs J. E. Kohler J. J. (2012). Sialidase specificity determined by chemoselective modification of complex sialylated glycans. ACS Chem. Biol. 7, 1509–1514. 10.1021/cb300241v 22704707 PMC3448839

[B120] Peri S. Kulkarni A. Feyertag F. Berninsone P. M. Alvarez-Ponce D. (2018). Phylogenetic Distribution of CMP-Neu5Ac Hydroxylase (CMAH), the Enzyme Synthetizing the Proinflammatory Human Xenoantigen Neu5Gc. Genome Biol. Evol. 10, 207–219. 10.1093/gbe/evx251 29206915 PMC5767959

[B121] Pericone C. D. Park S. Imlay J. A. Weiser J. N. (2003). Factors contributing to hydrogen peroxide resistance in Streptococcus pneumoniae include pyruvate oxidase (SpxB) and avoidance of the toxic effects of the fenton reaction. J. Bacteriol. 185, 6815–6825. 10.1128/JB.185.23.6815-6825.2003 14617646 PMC262707

[B122] Perrone L. A. Plowden J. K. Garcia-Sastre A. Katz J. M. Tumpey T. M. (2008). H5N1 and 1918 pandemic influenza virus infection results in early and excessive infiltration of macrophages and neutrophils in the lungs of mice. PloS Pathog. 4, e1000115. 10.1371/journal.ppat.1000115 18670648 PMC2483250

[B123] Pettigrew M. M. Marks L. R. Kong Y. Gent J. F. Roche-Hakansson H. Hakansson A. P. (2014). Dynamic changes in the Streptococcus pneumoniae transcriptome during transition from biofilm formation to invasive disease upon influenza A virus infection. Infect. Immun. 82, 4607–4619. 10.1128/IAI.02225-14 25135685 PMC4249342

[B124] Pittet L. A. Hall-Stoodley L. Rutkowski M. R. Harmsen A. G. (2010). Influenza virus infection decreases tracheal mucociliary velocity and clearance of Streptococcus pneumoniae. Am. J. Respir. Cell Mol. Biol. 42, 450–460. 10.1165/rcmb.2007-0417OC 19520922 PMC2848738

[B125] Plotkowski M. C. Puchelle E. Beck G. Jacquot J. Hannoun C. (1986). Adherence of type I Streptococcus pneumoniae to tracheal epithelium of mice infected with influenza A/PR8 virus. Am. Rev. Respir. Dis. 134, 1040–1044. 10.1164/arrd.1986.134.5.1040 3777666

[B126] Qin C. Zhou L. Hu Z. Zhang S. Yang S. Tao Y.. (2020). Dysregulation of Immune Response in Patients With Coronavirus 2019 (COVID-19) in Wuhan, China. Clin. Infect. Dis. 71, 762–768. 10.1093/cid/ciaa248 32161940 PMC7108125

[B127] Rawson T. M. Moore L. S. P. Zhu N. Ranganathan N. Skolimowska K. Gilchrist M.. (2020). Bacterial and Fungal Coinfection in Individuals With Coronavirus: A Rapid Review To Support COVID-19 Antimicrobial Prescribing. Clin. Infect. Dis. 71, 2459–2468. 10.1093/cid/ciaa530 32358954 PMC7197596

[B128] Reinoso-Vizcaino N. M. Cian M. B. Cortes P. R. Olivero N. B. Hernandez-Morfa M. Pinas G. E.. (2020). The pneumococcal two-component system SirRH is linked to enhanced intracellular survival of Streptococcus pneumoniae in influenza-infected pulmonary cells. PloS Pathog. 16, e1008761. 10.1371/journal.ppat.1008761 32790758 PMC7447016

[B129] Roberts S. Williams C. M. Salmon S. L. Bonin J. L. Metzger D. W. Furuya Y. (2019). Evaluation of Pneumococcal Surface Protein A as a Vaccine Antigen against Secondary Streptococcus pneumoniae Challenge during Influenza A Infection. Vaccines (Basel) 7 (4), 146. 10.3390/vaccines7040146 PMC6963301 31614565

[B130] Rodriguez A. E. Bogart C. Gilbert C. M. McCullers J. A. Smith A. M. Kanneganti T. D.. (2019). Enhanced IL-1beta production is mediated by a TLR2-MYD88-NLRP3 signaling axis during coinfection with influenza A virus and Streptococcus pneumoniae. PloS One 14, e0212236. 10.1371/journal.pone.0212236 30794604 PMC6386446

[B131] Rogers G. N. Paulson J. C. (1983). Receptor determinants of human and animal influenza virus isolates: differences in receptor specificity of the H3 hemagglutinin based on species of origin. Virology 127, 361–373. 10.1016/0042-6822(83)90150-2 6868370

[B132] Rowe H. M. Livingston B. Margolis E. Davis A. Meliopoulos V. A. Echlin H.. (2020). Respiratory Bacteria Stabilize and Promote Airborne Transmission of Influenza A Virus. mSystems 5 (5), e00762-20. 10.1128/mSystems.00762-20 32873612 PMC7470989

[B133] Rylance J. De Steenhuijsen Piters W. Mina M. J. Bogaert D. French N. Ferreira D. M.. (2019). Two Randomized Trials of the Effect of Live Attenuated Influenza Vaccine on Pneumococcal Colonization. Am. J. Respir. Crit. Care Med. 199, 1160–1163. 10.1164/rccm.201811-2081LE 30758980 PMC6515882

[B134] Sardu C. Gambardella J. Morelli M. B. Wang X. Marfella R. Santulli G. (2020). Hypertension, Thrombosis, Kidney Failure, and Diabetes: Is COVID-19 an Endothelial Disease? A Comprehensive Evaluation of Clinical and Basic Evidence. J. Clin. Med. 9 (5), 1417. 10.3390/jcm9051417 PMC7290769 32403217

[B135] Schauer R. Srinivasan G. V. Coddeville B. Zanetta J. P. Guerardel Y. (2009). Low incidence of N-glycolylneuraminic acid in birds and reptiles and its absence in the platypus. Carbohydr. Res. 344, 1494–1500. 10.1016/j.carres.2009.05.020 19541293

[B136] Schauer R. (2004). Sialic acids: fascinating sugars in higher animals and man. Zoology (Jena) 107, 49–64. 10.1016/j.zool.2003.10.002 16351927

[B137] Sender V. Hentrich K. Pathak A. Tan Qian Ler A. Embaie B. T. Lundstrom S. L.. (2020). Capillary leakage provides nutrients and antioxidants for rapid pneumococcal proliferation in influenza-infected lower airways. Proc. Natl. Acad. Sci. U.S.A. 117, 31386–31397. 10.1073/pnas.2012265117 33229573 PMC7733805

[B138] Shahangian A. Chow E. K. Tian X. Kang J. R. Ghaffari A. Liu S. Y.. (2009). Type I IFNs mediate development of postinfluenza bacterial pneumonia in mice. J. Clin. Invest. 119, 1910–1920. 10.1172/JCI35412 19487810 PMC2701856

[B139] Sharma-Chawla N. Sender V. Kershaw O. Gruber A. D. Volckmar J. Henriques-Normark B.. (2016). Influenza A Virus Infection Predisposes Hosts to Secondary Infection with Different Streptococcus pneumoniae Serotypes with Similar Outcome but Serotype-Specific Manifestation. Infect. Immun. 84, 3445–3457. 10.1128/IAI.00422-16 27647871 PMC5116722

[B140] Shinya K. Ebina M. Yamada S. Ono M. Kasai N. Kawaoka Y. (2006). Avian flu: influenza virus receptors in the human airway. Nature 440, 435–436. 10.1038/440435a 16554799

[B141] Short K. R. Habets M. N. Hermans P. W. Diavatopoulos D. A. (2012). Interactions between Streptococcus pneumoniae and influenza virus: a mutually beneficial relationship? Future Microbiol. 7, 609–624. 10.2217/fmb.12.29 22568716

[B142] Siegel S. J. Roche A. M. Weiser J. N. (2014). Influenza promotes pneumococcal growth during coinfection by providing host sialylated substrates as a nutrient source. Cell Host Microbe 16, 55–67. 10.1016/j.chom.2014.06.005 25011108 PMC4096718

[B143] Smith A. M. Adler F. R. Ribeiro R. M. Gutenkunst R. N. Mcauley J. L. McCullers J. A.. (2013). Kinetics of coinfection with influenza A virus and Streptococcus pneumoniae. PloS Pathog. 9, e1003238. 10.1371/journal.ppat.1003238 23555251 PMC3605146

[B144] Smith A. M. (2017). Quantifying the therapeutic requirements and potential for combination therapy to prevent bacterial coinfection during influenza. J. Pharmacokinet. Pharmacodyn. 44, 81–93. 10.1007/s10928-016-9494-9 27679506 PMC5376398

[B145] Snelgrove R. J. Goulding J. Didierlaurent A. M. Lyonga D. Vekaria S. Edwards L.. (2008). A critical function for CD200 in lung immune homeostasis and the severity of influenza infection. Nat. Immunol. 9, 1074–1083. 10.1038/ni.1637 18660812

[B146] Spelmink L. Sender V. Hentrich K. Kuri T. Plant L. Henriques-Normark B. (2016). Toll-Like Receptor 3/TRIF-Dependent IL-12p70 Secretion Mediated by Streptococcus pneumoniae RNA and Its Priming by Influenza A Virus Coinfection in Human Dendritic Cells. mBio 7, e00168–e00116. 10.1128/mBio.00168-16 26956584 PMC4810485

[B147] Springer S. A. Diaz S. L. Gagneux P. (2014). Parallel evolution of a self-signal: humans and new world monkeys independently lost the cell surface sugar Neu5Gc. Immunogenetics 66, 671–674. 10.1007/s00251-014-0795-0 25124893 PMC4198446

[B148] Stasiak A. C. Stehle T. (2020). Human adenovirus binding to host cell receptors: a structural view. Med. Microbiol. Immunol. 209, 325–333. 10.1007/s00430-019-00645-2 31784892 PMC7248032

[B149] Stegemann-Koniszewski S. Gereke M. Orrskog S. Lienenklaus S. Pasche B. Bader S. R.. (2013). TLR7 contributes to the rapid progression but not to the overall fatal outcome of secondary pneumococcal disease following influenza A virus infection. J. Innate Immun. 5, 84–96. 10.1159/000345112 23154432 PMC6741512

[B150] Stevens J. Blixt O. Paulson J. C. Wilson I. A. (2006). Glycan microarray technologies: tools to survey host specificity of influenza viruses. Nat. Rev. Microbiol. 4, 857–864. 10.1038/nrmicro1530 17013397 PMC7097745

[B151] Stock J. B. Ninfa A. J. Stock A. M. (1989). Protein phosphorylation and regulation of adaptive responses in bacteria. Microbiol. Rev. 53, 450–490. 10.1128/MR.53.4.450-490.1989 2556636 PMC372749

[B152] Sun K. Metzger D. W. (2008). Inhibition of pulmonary antibacterial defense by interferon-gamma during recovery from influenza infection. Nat. Med. 14, 558–564. 10.1038/nm1765 18438414

[B153] Sun J. Madan R. Karp C. L. Braciale T. J. (2009). Effector T cells control lung inflammation during acute influenza virus infection by producing IL-10. Nat. Med. 15, 277–284. 10.1038/nm.1929 19234462 PMC2693210

[B154] Sun K. Ye J. Perez D. R. Metzger D. W. (2011). Seasonal FluMist vaccination induces cross-reactive T cell immunity against H1N1 (2009) influenza and secondary bacterial infections. J. Immunol. 186, 987–993. 10.4049/jimmunol.1002664 21160043

[B155] Suzuki Y. (2005). Sialobiology of influenza: molecular mechanism of host range variation of influenza viruses. Biol. Pharm. Bull. 28, 399–408. 10.1248/bpb.28.399 15744059

[B156] Taubenberger J. K. Morens D. M. (2020). The 1918 Influenza Pandemic and Its Legacy. Cold Spring Harb. Perspect. Med. 10 (10), a038695. 10.1101/cshperspect.a038695 31871232 PMC7528857

[B157] Throup J. P. Koretke K. K. Bryant A. P. Ingraham K. A. Chalker A. F. Ge Y.. (2000). A genomic analysis of two-component signal transduction in Streptococcus pneumoniae. Mol. Microbiol. 35, 566–576. 10.1046/j.1365-2958.2000.01725.x 10672179

[B158] Trappetti C. Kadioglu A. Carter M. Hayre J. Iannelli F. Pozzi G.. (2009). Sialic acid: a preventable signal for pneumococcal biofilm formation, colonization, and invasion of the host. J. Infect. Dis. 199, 1497–1505. 10.1086/598483 19392624

[B159] Van Der Sluijs K. F. Van Elden L. J. Nijhuis M. Schuurman R. Pater J. M. Florquin S.. (2004). IL-10 is an important mediator of the enhanced susceptibility to pneumococcal pneumonia after influenza infection. J. Immunol. 172, 7603–7609. 10.4049/jimmunol.172.12.7603 15187140

[B160] Van Der Sluijs K. F. Nijhuis M. Levels J. H. Florquin S. Mellor A. L. Jansen H. M.. (2006a). Influenza-induced expression of indoleamine 2,3-dioxygenase enhances interleukin-10 production and bacterial outgrowth during secondary pneumococcal pneumonia. J. Infect. Dis. 193, 214–222. 10.1086/498911 16362885

[B161] Van Der Sluijs K. F. Van Elden L. J. Nijhuis M. Schuurman R. Florquin S. Shimizu T.. (2006b). Involvement of the platelet-activating factor receptor in host defense against Streptococcus pneumoniae during postinfluenza pneumonia. Am. J. Physiol. Lung Cell Mol. Physiol. 290, L194–L199. 10.1152/ajplung.00050.2005 16100290

[B162] Van Someren Greve F. Van Der Sluijs K. F. Tuip A. M. Schultz M. J. De Jong M. D. Juffermans N. P. (2018). Treatment with broadly neutralizing influenza antibodies reduces severity of secondary pneumococcal pneumonia in mice. J. Med. Virol. 90, 1431–1437. 10.1002/jmv.25212 29718555 PMC6055667

[B163] Vaughn V. M. Gandhi T. Petty L. A. Patel P. K. Prescott H. C. Malani A. N.. (2020). Empiric Antibacterial Therapy and Community-onset Bacterial Co-infection in Patients Hospitalized with COVID-19: A Multi-Hospital Cohort Study. Clin. Infect. Dis. ciaa1239 10.1093/cid/ciaa1239 32820807 PMC7499526

[B164] Verma A. K. Bansal S. Bauer C. Muralidharan A. Sun K. (2020). Influenza Infection Induces Alveolar Macrophage Dysfunction and Thereby Enables Noninvasive Streptococcus pneumoniae to Cause Deadly Pneumonia. J. Immunol. 205, 1601–1607. 10.4049/jimmunol.2000094 32796026 PMC7484308

[B165] Von Itzstein M. Wu W. Y. Kok G. B. Pegg M. S. Dyason J. C. Jin B.. (1993). Rational design of potent sialidase-based inhibitors of influenza virus replication. Nature 363, 418–423. 10.1038/363418a0 8502295

[B166] World Health Organization (2017). The Global Impact of Respiratory Disease. Available at: https://www.who.int/gard/publications/The_Global_Impact_of_Respiratory_Disease.pdf (Accessed Dec 15 2020).

[B167] World Health Organization (2018). Influenza (Seasonal). Available at: https://www.who.int/news-room/fact-sheets/detail/influenza-(seasonal) (Accessed Dec 15 2020).

[B168] World Health Organization (2019). Ten threats to global health in 2019. Available at: https://www.who.int/news-room/spotlight/ten-threats-to-global-health-in-2019 (Accessed Feb 09 2021).

[B169] World Health Organization (2020). WHO Coronavirus Disease (COVID-19) Dashboard. Available at: https://covid19.who.int/ (Accessed Feb 09 2021).

[B170] Wren J. T. Blevins L. K. Pang B. Basu Roy A. Oliver M. B. Reimche J. L.. (2017). Pneumococcal Neuraminidase A (NanA) Promotes Biofilm Formation and Synergizes with Influenza A Virus in Nasal Colonization and Middle Ear Infection. Infect. Immun. 85 (4), e01044-16. 10.1128/IAI.01044-16 28096183 PMC5364304

[B171] Wu Y. Mao H. Ling M. T. Chow K. H. Ho P. L. Tu W.. (2011). Successive influenza virus infection and Streptococcus pneumoniae stimulation alter human dendritic cell function. BMC Infect. Dis. 11, 201. 10.1186/1471-2334-11-201 21771345 PMC3146832

[B172] Wu M. Gibbons J. G. Deloid G. M. Bedugnis A. S. Thimmulappa R. K. Biswal S.. (2017). Immunomodulators targeting MARCO expression improve resistance to postinfluenza bacterial pneumonia. Am. J. Physiol. Lung Cell Mol. Physiol. 313, L138–L153. 10.1152/ajplung.00075.2017 28408365 PMC5538876

[B173] Xu G. Suzuki T. Maejima Y. Mizoguchi T. Tsuchiya M. Kiso M.. (1995). Sialidase of swine influenza A viruses: variation of the recognition specificities for sialyl linkages and for the molecular species of sialic acid with the year of isolation. Glycoconj. J. 12, 156–161. 10.1007/BF00731360 7620333

[B174] Yamada S. Suzuki Y. Suzuki T. Le M. Q. Nidom C. A. Sakai-Tagawa Y.. (2006). Haemagglutinin mutations responsible for the binding of H5N1 influenza A viruses to human-type receptors. Nature 444, 378–382. 10.1038/nature05264 17108965

[B175] Yan R. Zhang Y. Li Y. Xia L. Guo Y. Zhou Q. (2020). Structural basis for the recognition of SARS-CoV-2 by full-length human ACE2. Science 367, 1444–1448. 10.1126/science.abb2762 32132184 PMC7164635

[B176] Zhang B. Zhou X. Qiu Y. Song Y. Feng F. Feng J.. (2020). Clinical characteristics of 82 cases of death from COVID-19. PloS One 15, e0235458. 10.1371/journal.pone.0235458 32645044 PMC7347130

[B177] Zhao H. To K. K. W. Sze K. H. Yung T. T. Bian M. Lam H.. (2020). A broad-spectrum virus- and host-targeting peptide against respiratory viruses including influenza virus and SARS-CoV-2. Nat. Commun. 11, 4252. 10.1038/s41467-020-17986-9 32843628 PMC7447754

[B178] Zhou F. Yu T. Du R. Fan G. Liu Y. Liu Z.. (2020). Clinical course and risk factors for mortality of adult inpatients with COVID-19 in Wuhan, China: a retrospective cohort study. Lancet 395, 1054–1062. 10.1016/S0140-6736(20)30566-3 32171076 PMC7270627

[B179] Zhu X. Ge Y. Wu T. Zhao K. Chen Y. Wu B.. (2020). Co-infection with respiratory pathogens among COVID-2019 cases. Virus Res. 285, 198005. 10.1016/j.virusres.2020.198005 32408156 PMC7213959

[B180] Zimmer G. Suguri T. Reuter G. Yu R. K. Schauer R. Herrler G. (1994). Modification of sialic acids by 9-O-acetylation is detected in human leucocytes using the lectin property of influenza C virus. Glycobiology 4, 343–349. 10.1093/glycob/4.3.343 7949660 PMC7108540

